# Effects of diabetes on microglial physiology: a systematic review of in vitro, preclinical and clinical studies

**DOI:** 10.1186/s12974-023-02740-x

**Published:** 2023-03-03

**Authors:** María Vargas-Soria, Mónica García-Alloza, Miriam Corraliza-Gómez

**Affiliations:** 1 Division of Physiology, School of Medicine, Universidad de Cadiz, Cadiz, Spain; 2 Instituto de Investigacion e Innovacion en Ciencias Biomedicas de la Provincia de Cadiz (INIBICA), Cadiz, Spain

**Keywords:** Microglia, Diabetes mellitus, Neuroinflammation, Cytokines, Brain, Retina, Hyperglycemia, Oxidative stress

## Abstract

Diabetes mellitus is a heterogeneous chronic metabolic disorder characterized by the presence of hyperglycemia, commonly preceded by a prediabetic state. The excess of blood glucose can damage multiple organs, including the brain. In fact, cognitive decline and dementia are increasingly being recognized as important comorbidities of diabetes. Despite the largely consistent link between diabetes and dementia, the underlying causes of neurodegeneration in diabetic patients remain to be elucidated. A common factor for almost all neurological disorders is neuroinflammation, a complex inflammatory process in the central nervous system for the most part orchestrated by microglial cells, the main representatives of the immune system in the brain. In this context, our research question aimed to understand how diabetes affects brain and/or retinal microglia physiology. We conducted a systematic search in PubMed and Web of Science to identify research items addressing the effects of diabetes on microglial phenotypic modulation, including critical neuroinflammatory mediators and their pathways. The literature search yielded 1327 records, including 18 patents. Based on the title and abstracts, 830 papers were screened from which 250 primary research papers met the eligibility criteria (original research articles with patients or with a strict diabetes model without comorbidities, that included direct data about microglia in the brain or retina), and 17 additional research papers were included through forward and backward citations, resulting in a total of 267 primary research articles included in the scoping systematic review. We reviewed all primary publications investigating the effects of diabetes and/or its main pathophysiological traits on microglia, including in vitro studies, preclinical models of diabetes and clinical studies on diabetic patients. Although a strict classification of microglia remains elusive given their capacity to adapt to the environment and their morphological, ultrastructural and molecular dynamism, diabetes modulates microglial phenotypic states, triggering specific responses that include upregulation of activity markers (such as Iba1, CD11b, CD68, MHC-II and F4/80), morphological shift to amoeboid shape, secretion of a wide variety of cytokines and chemokines, metabolic reprogramming and generalized increase of oxidative stress. Pathways commonly activated by diabetes-related conditions include NF-κB, NLRP3 inflammasome, fractalkine/CX3CR1, MAPKs, AGEs/RAGE and Akt/mTOR. Altogether, the detailed portrait of complex interactions between diabetes and microglia physiology presented here can be regarded as an important starting point for future research focused on the microglia–metabolism interface.

**Supplementary Information:**

The online version contains supplementary material available at 10.1186/s12974-023-02740-x.

## Introduction

Microglia are the highly heterogeneous and long-lived resident macrophages of the central nervous system (CNS) and maintain brain homeostasis by constantly surveying the environment. These immunocompetent cells act as professional phagocytes and orchestrators of the innate immune response to protect the brain against pathogenic factors [1]. To accomplish their functions, microglia are extraordinarily responsive to any alteration in their environment, and consequently their morphology and molecular profile are highly dynamic and plastic, resulting in many diverse and context-dependent phenotypes or functional states [2]. “Diabetes mellitus” (DM) describes a group of metabolic disorders characterized by the presence of hyperglycemia. DM is a group of chronic diseases that occur either when the pancreas does not produce enough of the hormone insulin, or when the body cannot effectively use the insulin it produces [3]. Age remains a major risk factor for developing dementia and DM; therefore, the progressive rise of life expectancy is secondarily contributing to an increase in the number of people living with dementia [4] or diabetes and diabetes-related complications [5]. In this sense, hyperinsulinemia and glucose intolerance could contribute to neurodegeneration. Furthermore, metabolic disturbances have been reported to induce low-grade chronic inflammation, which can ultimately trigger neuroinflammation. The number of records covering the relationship between diabetes and microglia has exponentially increased in recent years; however, to the best of our knowledge no previous review has synthesized the existing evidence about the effects of DM on microglial physiology following systematic search methods. As such, the present systematic review aims to summarize the knowledge concerning diabetes-induced microglial phenotypes, and to discuss the implications of these findings in the brain and the retina.

## Methods

The objective of this systematic review was to examine the effects of diabetes on microglial cells. To that end, we conducted a systematic literature review of primary research papers (published between database inception and February 28th, 2022) according to the recommendations of the Preferred Reporting Items for Systematic Reviews and Meta-Analyses (PRISMA) guidelines [6]. The systematic search was based on the Population, Intervention, Comparison and Outcome (i.e., “PICO”) strategy, investigating whether microglia (population) subjected to diabetes-related conditions (intervention) show differential phenotypic modulation (outcome) when compared with microglia in a non-diabetic environment (comparison). These aspects were used as a guide to formulate the focused research question—how does diabetes (I) affect microglia (P) phenotypes (O)? A comprehensive search was performed on PubMed and Web of Science databases, using the search query “((microglia) AND (diabetes OR hyperglycemia))”. Inclusion and exclusion criteria were defined and agreed by all authors. Studies were included if they fulfilled the following eligibility criteria: (1) original full-text articles; (2) written in English; (3) focused on diabetes and (4) contained data about brain and/or retinal microglia. Furthermore, additional articles identified through forward and backward citations in the studies found by the search algorithm were also included. Reasons for exclusion of an article were: (1) written in other languages (besides English); (2) not primary research articles (including reviews, medical/meeting abstracts, patents and tools/methodological reports); (3) retracted publications; (4) inaccessible complete manuscripts; (5) studies on diabetic comorbidities, including macrovascular, microvascular and neurodegenerative diseases.

For study selection process, a cross-validation of the protocol was performed in which each author independently screened titles and abstracts of a random sample of 50 records and discussed inconsistencies until consensus was obtained. Finally, one of the authors screened titles and abstracts for each record and decided which articles to include. If necessary, the other two authors were consulted to make the final decision about an article.

According to the abstract information, articles were tagged into the following categories: in vitro, preclinical models or clinical studies. Following full-text article reading, we extracted data from eligible studies and classified each tagged report with subheading terms to organize the review structure. We collected data on the report (author, year and source of publication), the study (sample characteristics) and the research design (experimental paradigm). All data were synthesized in Additional file 1: Table S1. Due to the fact that some steps were not totally performed dually and independently by more than one reviewer, it is possible that a small risk of bias was introduced in studies and data selection. Nonetheless, articles were cross validated, and therefore, this methodological limitation should not modify the overall conclusions of this literature review. All articles which met the criteria were included and the quality of the articles was assumed, given the peer-reviewed process followed by primary research articles. While not performing a quality control assessment might be limiting, the complex and multifactorial approaches of all included studies reduces this possibility. Ultimately, this study might be regarded as an important starting point for researchers focused on the complex relationships between microglia and diabetes in the future.

## Results

### Description of studies included and basic concepts about microglia

**Fig. 1:**
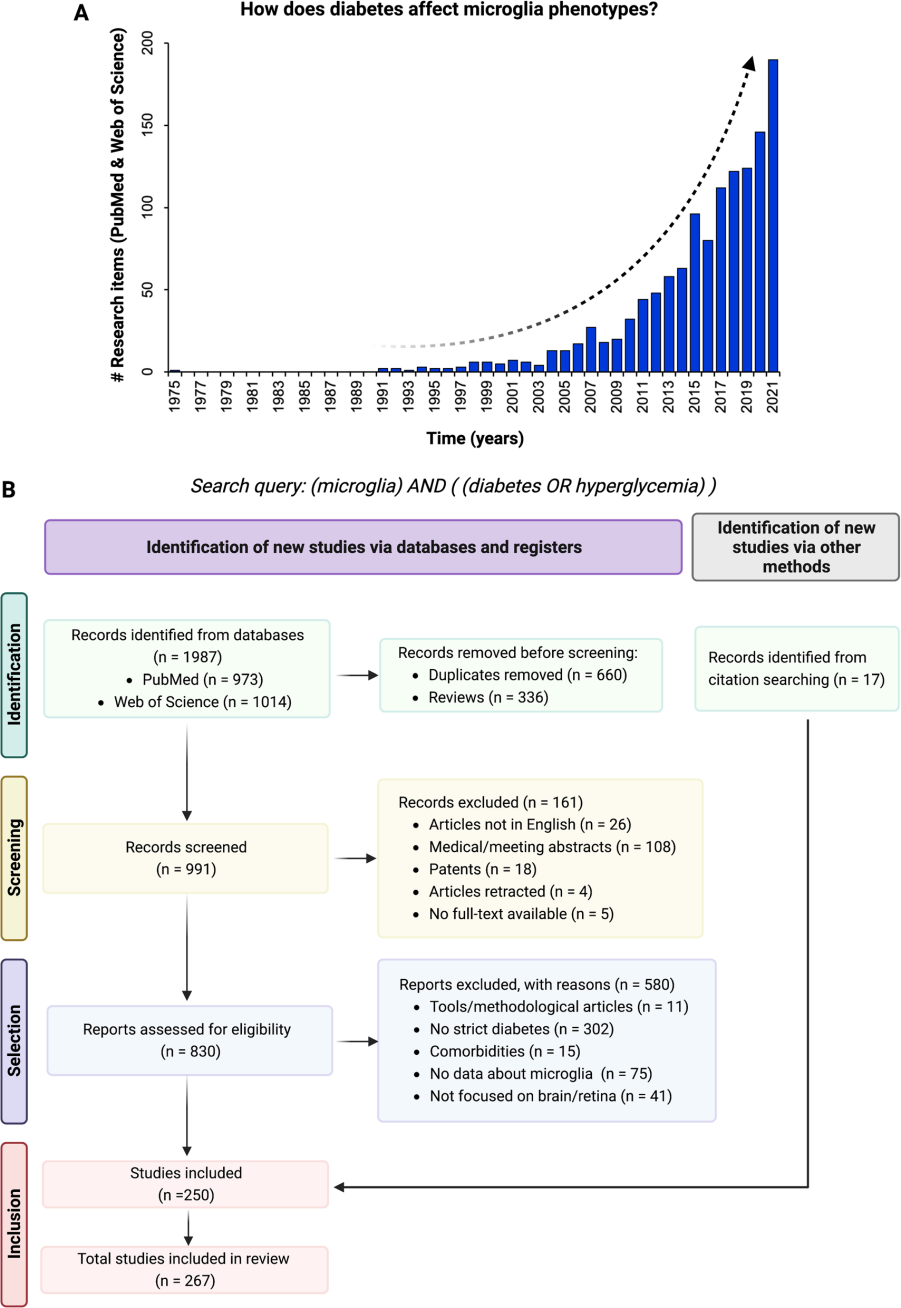
Literature search and inclusion criteria. **a** Timeline of research items recovered by PubMed and Web of Science using the search query defined for our systematic review. **b** PRISMA 2020 flow diagram

Our research question identified a total of 1987 records published between 1975 and 2022, showing a clear exponential growth in the number of studies addressing the complex relationship between microglia and diabetes (Fig. 1a). Before screening, studies in duplicate (*n* = 662) and narrative reviews (*n* = 336) were removed, resulting in selection of 991 research items. After first title/abstract screening, 161 records were excluded according to the exclusion criteria, resulting in selection of 830 papers for further assessment. Following inclusion criteria, 250 studies met the eligibility criteria and were tagged for review. Furthermore, 17 additional research papers were added through forward and backward citations, resulting in a total of 267 studies, which were stored in a Zotero collection (available in Additional file 1: Table S1). Figure 1b summarizes the systematic review process workflow.

Microglia, the most dynamic cells of the healthy mature brain, exist in diverse multidimensional states depending on the context and local environment. Microglial states, modulated by the spatiotemporal context, are defined by a set of variables (including cellular processes, molecular markers, inflammatory mediators, molecular pathways and cell morphology) driving phenotypic transformations of microglia in response to a specific stimulus or condition [2]. The term “homeostatic” is used to refer to microglia in a physiological context, as opposed to “activated” microglia—a broad term that encompasses a great variety of responses toward diverse stimuli, including stress, inflammation and neurodegenerative conditions. In this review we will avoid this terminology and refer instead to the specific stimulus-induced responses. Morphological changes were previously used to assess functional state, in which “homeostatic” microglia show fusiform cell bodies with long and thin branching processes, whereas upon stimulation microglia undergo a morphological shift toward a “responsive” phenotype, characterized by an amoeboid shape with enlarged cell body and decreased branching with thickened processes [7]. Despite the fact that it is considered that morphological changes should not be equated with function, they add relevant information on microglial phenotype [2]. Following recent recommendations for microglia researchers [2], we have summarized all current evidence regarding microglia responses to diabetic conditions using as many as possible layers of complexity (from in vitro studies mimicking relevant diabetes-related conditions to preclinical models of diabetes and clinical studies on diabetic patients) to get a complete portrait of microglia in the diabetic environment.

### In vitro studies

**Fig. 2:**
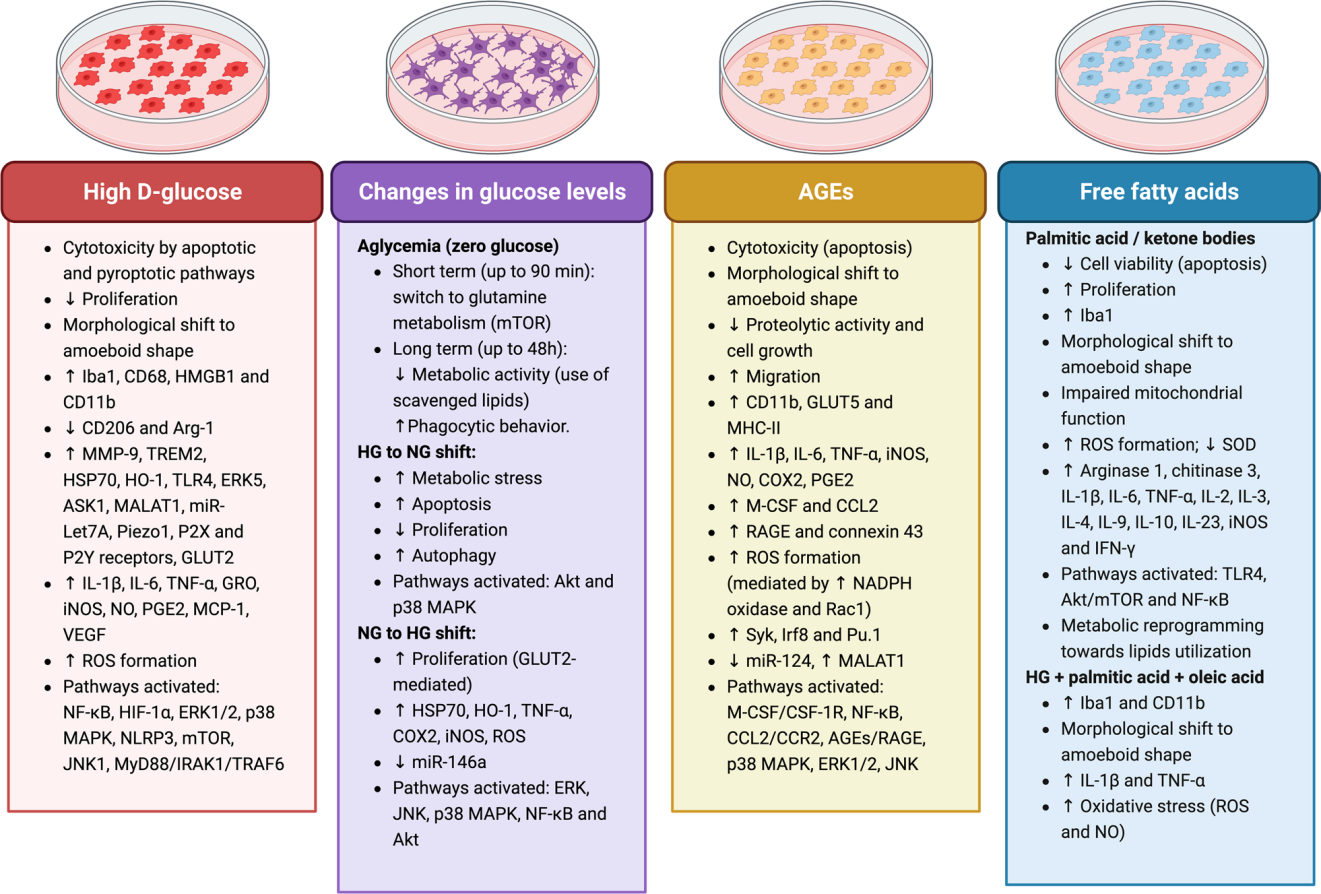
Microglia responses to diabetes-related conditions in vitro. Responses of cell cultures to high d-glucose, changes in glucose levels, advanced glycation end products (AGEs) and free fatty acids are summarized

In this section, we will analyze the main experimental strategies to mimic a diabetic environment in cell cultures, as well as the microglial phenotypes triggered by such conditions. Figure 2 contains a summary of described microglial responses to diabetes-related conditions in vitro.

#### High glucose

The brain uses glucose as a primary energy source, thus any type of dysglycemia (including hyperglycemia, hypoglycemia, and acute glycemic fluctuations) may underlie cerebral complications in diabetic patients. Glucose plays a crucial role in maintaining the metabolism of immune cells, and it is also essential to modulate the immune response. In fact, stimulus-responding immune cells are highly dependent on glucose, not only to maintain anaerobic ATP production but also to serve as an anabolic substrate. Many studies have addressed the effects of high d-glucose (HG) exposure on different aspects of microglial physiology. Exposure of microglia to HG for 24 h triggers a dose-dependent cytotoxicity [8–12] through augmented caspase-1 activity [12] and increased apoptosis [11], the latter probably mediated, at least in part, by apoptosis signal-regulating kinase 1 (ASK1), which is overexpressed upon HG conditions [8]. Another study reported that HG activates pyroptosis, an inflammatory type of cell death, by upregulating the expression of NLR family pyrin domain containing 3 (NLRP3), cleaved caspase-1 and cleaved gasdermin D [12]. Another cellular process affected by HG exposure is proliferation, which is decreased upon glucose-induced stress [9, 13]. Furthermore, several studies agree that, upon HG stimulation, microglia undergo a morphological shift from a ramified shape to amoeboid-like morphology [9, 14–19].

Despite the great variability among studies, including differences in glucose concentration, exposure time and cell type, many approaches concur that HG treatment triggers the overexpression of classically considered “pro-inflammatory” markers, including ionized calcium-binding adapter molecule 1 (Iba1) [12, 20–24], cluster of differentiation (CD)68 [8, 13]; high mobility group box-1 protein (HMGB1) [17, 25] and CD11b [15, 26], and concomitantly downregulates the expression of “anti-inflammatory” microglial markers, such as CD206 [8, 17] and arginase-1 [26]. Interestingly, ASK1 increases CD68, tumor necrosis factor alpha (TNF-α) and IL-6 expression while concomitantly decreases CD206 and IL-10 expression [8]. Moreover, HG-triggered ASK1 activation promotes miR-Let7A expression and, conversely, Let7A reduces ASK1 activation, exerting an “anti-inflammatory like” effect on microglia by reverting ASK1-mediated N-Myc and c-Myc activation [8]. Accordingly, HG exposure on microglia upregulates pro-inflammatory mediators, such as TNF-α [8, 11, 15, 17, 18, 21–24, 26, 26–28]; IL-6 [8, 11, 15, 22, 23, 26, 29, 30], IL-1β [11, 12, 17, 18, 21–23, 28, 31, 32]; interferon-gamma (IFN-γ) [30]; GRO, which is a rat ortholog of human IL-8 [11, 14]; inducible nitric oxide synthase (iNOS) [18, 26]; nitric oxide (NO) and prostaglandin E2 [18] and monocyte chemoattractant protein 1 (MCP-1, also referred to as CCL2) [27]. Remarkably, exposure of human microglia to chronic long-term hyperglycemia (up to 12 days in culture) revealed that dynamic changes of microglial phenotypes are mediated by extracellular signal-regulated kinase (ERK)5 signaling [33]. In this transition, microglia undergo three polarization stages (at the mRNA level): at 2 days arginase-1, CD206 and IL-10 are induced, while “pro-inflammatory” markers are absent; from 4 to 6 days, “anti-inflammatory” markers are still expressed but in a lesser extent, while iNOS, TNF-α, IL-6 and IL-12 are upregulated; from 8 days, arginase-1, CD206 and IL-10 drop and are almost abolished, while “pro-inflammatory” markers remain elevated [33].

Regarding the pathways activated by d-glucose, HG induces reactive oxygen species (ROS) formation [14, 16, 23, 27] and subsequent activation of oxidative-stress induced pathways, including protein kinase C [14] and nuclear factor kappa B (NF-κB) [27], whose activation result in inflammatory responses. In fact, HG activates the NF-κB pathway at different levels: toll-like receptor 4 (TLR4) upregulation [18], increased phosphorylation of NF-κB p65 subunit [15, 23], IKK and IκB (inhibitors of NF-κB kinase) [22], and nuclear translocation from cytosol to nucleus of p65 [18, 21, 22, 25] and early growth response protein 1 [23]. Accordingly, HG induces matrix metalloproteinase-9 overexpression via HMGB1/TLR4/p38/NF-κB signaling pathway [25]. In addition, HG treatment activates mitogen-activated protein kinase (MAPK) cascades, including p38 MAPK pathway [25], ERK1/2 phosphorylation [21–24], as well as its upstream kinases cRaf and mitogen-activated protein kinase kinase (MEK)1/2 [21, 23, 24]. HG induces hypoxia-inducible factor 1-alpha (HIF-1α) translocation into the nucleus, where it acts as a master transcription factor for regulating the expression of vascular endothelial growth factor (VEGF) [20, 34], and such activation is mediated by ERK1/2 pathway [34]. HG also triggers calcium peaks in microglia by two different mechanisms, namely, activation of P2X receptors [35] and overexpression of the mechanically sensitive cation channel Piezo1 [9], which increase both the influx of extracellular and the internal-stored calcium release into the cytosol, probably due to P2Y receptor activation [9, 35]. Such calcium peaks result in microglial damage through the activation of mammalian target of rapamycin (mTOR) and Jun N-terminal kinase (JNK)1 [9]. In line with JNK activation, HG also upregulates other stress-inducible proteins including 70 kilodalton heat shock protein and heme oxygenase 1 [36]. HG exposure increases triggering receptor expressed on myeloid cells 2 (TREM2) transcription and expression levels as soon as 8 h after glucose exposure, and such increase is maintained until 48 h of treatment. Concomitantly, NLRP3 is also overexpressed upon HG conditions [32]. Removal of either TREM2 or NLRP3 by CRISPR technology results in decreased IL-1β expression and secretion, indicating that TREM2 promotes HG-induced microglial inflammation by interacting with the NLRP3 inflammasome [32]. Furthermore, HG promotes the overexpression of the long non-protein coding RNA metastasis-associated lung adenocarcinoma transcript 1 (MALAT1), which acts as a positive regulator of the expression of myeloid differentiation primary response 88 (MyD88) by increasing the level of H3 histone acetylation [11]. Consequently, downstream proteins such as interleukin 1 receptor-associated kinase 1 (IRAK1) and tumor necrosis factor receptor-associated factor 6 (TRAF6) are also upregulated [11].

Glucose uptake in microglia is mainly mediated by glucose transporter type 1 (GLUT1) [24]. The effects of HG on GLUT1 expression are still controversial: a recent work described that with increasing glucose concentration, GLUT1 mRNA is decreased [37], whereas other study reported that GLUT1 mRNA is increased upon HG exposure, and such overexpression is mediated by ERK1/2 and NF-κB activation [23]. Conversely, GLUT1 protein remains unchanged upon HG treatment [36]. On the other hand, glucose transporter type 2 (GLUT2) is upregulated upon HG exposure, and it is involved in enhanced glucose uptake and utilization and increased activity of the mitochondrial respiratory chain [36].

Pre-exposure to HG levels has consequences on other microglial functions, such as lipopolysaccharide (LPS) response. In this regard, 1 h pre-exposure to HG levels triggers microglial priming, making microglia more vulnerable to LPS-induced apoptosis, and such synergistic cytotoxicity is mediated by oxidative radicals [10]. Similar synergistic effects are observed in terms of inflammatory response, since microglia cultured in HG show higher LPS-induced TNF-α, IL-6, iNOS and nitrite production [28, 37, 38] compared to LPS-stimulated cells cultured under normal glucose conditions, as well as decreased arginase-1, IL-4 and IL-10 expression [38], indicative of an exacerbated pro-inflammatory polarization. The proposed mechanisms for LPS-induced HG-enhanced inflammatory responses in microglia are the TLR4/Janus kinase 2 (JAK2)/Signal transducer and activator of transcription 3 (STAT3) pathway [28], as well as the TLR4/MyD88/NF-κB pathway [38].

#### Changes in glucose levels

Acute glycemic variability may also be responsible for brain alterations associated with diabetes, since glucose is the main source of metabolic fuel for the brain.

Microglia are highly glycolytic under homeostatic conditions, metabolizing glucose through the tricarboxylic acid cycle to support mitochondrial oxidative phosphorylation [39]. However, during short term (up to 90 min) aglycemia (0 mM glucose) glucose-deprived microglia show a unique ability to maintain mitochondrial activity using glutamine as an alternative carbon source. This rapid switch to glutamine metabolism is mTOR-mediated and allows microglia to perform surveillant functions even in the absence of glucose [39]. Prolonged aglycemia (up to 48 h) results in decreased cell metabolic activity, resulting in decreased oxidative phosphorylation and activity of the pentose phosphate pathway, with scavenged lipids used as an alternative energy source during metabolic stress [40]. Glucose deprivation does not affect either viability or proliferation, but it triggers phagocytic behavior in microglia, which in the absence of glucose increases surveillance functions [40]. Glucose deprivation per se does not elicit the release of inflammatory mediators. The effects of glucose deprivation on LPS-induced microglial phenotype remain to be elucidated. One study described a priming effect by which glucose-deprived microglia show a higher response to LPS treatment [40]. However, another reported that 2-deoxyglucose, a competitive inhibitor of d-glucose metabolism, suppresses LPS-induced inflammatory response by decreasing the NADH:NAD^+^ ratio, whereas lactate supplementation restores the inflammatory response to LPS [41]. In line with this, glucose flux through the hexose monophosphate shunt is required for LPS-induced NO production [42]. Collectively, microglia show a unique metabolic flexibility and reprogramming to adapt to glucose deprivation.

Both upward and downward acute changes in glycemia also affect microglia activity. Chronic normal d-glucose (NG) to HG shift increases proliferation mediated by GLUT2, which is overexpressed compared with cells under constant NG. Conversely, HG to NG shift creates a metabolic stress that promotes downregulation of energy-consuming processes such as proliferation, concomitant with a stimulation of catabolic processes like autophagy, accompanied by increased apoptosis (decreased Bcl-2 and increased cleaved caspase-3 protein levels) [36]. Hyperglycemic shift upregulates stress- and inflammatory-related proteins, including 70 kilodalton heat shock protein, heme oxygenase 1, TNF-α, cyclooxygenase-2, iNOS and oxygen free radicals, whereas a shift from HG to NG reduces these HG-mediated effects [36]. Interestingly, NG to HG-induced microglia polarization is mediated by miR-146a, which is significantly decreased in comparison with the NG control group [26]. In fact, miR-146a overexpression has anti-inflammatory effects on the NG to HG microglia by inhibiting activation of the NF-κB pathway [26]. Acute NG to HG shift rapidly activates intracellular signaling cascades, including ERK, JNK, p38, NF-κB and Akt pathways, while the HG to NG shift only activates Akt and p38 signaling [36].

#### Advanced glycation end products

Advanced glycation end products (AGEs) are proteins or lipids that become glycated as a result of exposure to HG levels and constitute a heterogeneous population of highly reactive compounds. Due to exposure to chronically elevated blood glucose levels, diabetic patients show increased accumulation of Amadori-glycated proteins, generated by the condensation of one or more sugar aldehydes with free amino groups on proteins. Such Amadori-glycated proteins may undergo further irreversible modifications to form AGEs.

A study comparing human brain cell types revealed that the toxic AGE–albumin compound is mainly produced by microglia, and the rate of AGE–albumin synthesis is increased upon oxidative stress [43]. AGEs treatment results in both the uptake of AGEs by microglia and a concomitant decrease in cell viability [44, 45]. Interestingly, high d-glucose exposure of insulin causes insulin glycation, and such glycated insulin triggers apoptosis-mediated cytotoxicity in microglia [46]. AGEs also decrease proteolytic microglial activity (both proteasomal and lysosomal pathways) [44] and inhibit cell growth [47].

Microglia exposure to AGEs triggers a dose- and time-dependent shift in morphology, from ramified to amoeboid forms [19, 45, 48–51], and increases microglial migration [52]. AGEs-induced microglial phenotypic modulation is supported by the overexpression of markers of microglial activity, such as CD11b [44, 45, 48], Iba1 [50], glucose transporter type 5 (GLUT5) and major histocompatibility complex II (MHC-II) [53], as well as the overproduction of inflammatory mediators: IL-1β [19, 45, 50, 51, 54], IL-6 [51, 54], TNF-α [19, 45, 48–52, 54, 55], cyclooxygenase-2 and prostaglandin E2 [45], iNOS and NO [51, 56]. AGEs exposure also promotes NADPH oxidase 2 expression [45] and increases ROS production in a dose- and time-dependent manner [45, 47, 48, 54, 55], along with increased lipid peroxidation [45] and reduced cellular anti-oxidative capacity [45, 48]. Concomitant with decreased glutathione content and superoxide dismutase activity, AGEs also stimulate the nuclear factor erythroid 2-related factor 2 (Nrf2) antioxidant system by the overexpression of Nrf2 and heme oxygenase 1, although it is insufficient to prevent AGEs-induced oxidative stress [45].

Regarding signaling pathways, AGEs promote the expression of different molecules that modulate microglial activity, including macrophage colony-stimulating factor (M-CSF) [50] and chemokine CCL2 [19]. M-CSF acts as a co-stimulator of AGEs involved in the release of inflammatory factors through the colony stimulating factor 1 receptor (CSF1R) signaling pathway [50]. In this context, spleen tyrosine kinase (Syk) protein is a relevant mediator of AGEs-induced microglial inflammatory response through the upregulation of transcription factors Irf8 and Pu.1 [19]. In neuron–microglia co-cultures exposed to AGEs, neurons are the main source of MCP-1, and this molecule has a significant effect on microglia phenotype, increasing CD11b expression, augmenting migration and promoting TNF-α secretion through the activation of C–C motif chemokine receptor (CCR)2 in microglia [57, 58]. Downstream pathways activated in microglia by AGEs-induced MCP-1 release include p38 MAPK, ERK and NF-κB [58]. AGEs also induce microglial reactivity via NF-κB and MAPK signaling pathways [48, 49, 51, 57, 58]. Specifically, AGEs increase phosphorylation of IκBα (NF-κB inhibitor, alpha) [49], promote the translocation of NF-κB p65 into the nucleus [48, 49, 58], and enhance NF-κB DNA binding activity and transcription of many target genes including TNF-α, IL-1β, intercellular adhesion molecule 1 (ICAM-1) and the receptor for advanced glycation end products (RAGE) [49]. The MAPK pathways ERK1/2 [48, 58], p38 MAPK [48, 49, 51, 58], JNK [49, 51] and c-Jun [48] are also stimulated by AGEs exposure, resulting in the overproduction of inflammatory mediators. In addition, RAGE is overexpressed by microglia in response to AGEs exposure [45, 53]. Activation of the AGE/RAGE signaling pathway by AGEs results in increased TNF-α secretion, accompanied by augmented TNF-receptor type II expression [53]. AGEs-mediated TNF-α signaling in microglia results in the overexpression of connexin 43, as well as gap junction/hemichannel opening, indicating increased microglial cell communication [53]. Interestingly, AGEs treatment not only activates the RAGE pathway, but also triggers the recruitment of signal transducers of the AGE/RAGE system, including RAGE itself and the tyrosine kinase Src, into lipid rafts [49]. Furthermore, pre-stimulation with AGEs potentiates LPS-induced microglial response and increases NO and CD11b production [44].

AGEs exposure also has effect on microglial non-coding RNAs. In this way, AGEs treatment significantly downregulates the expression of miR-124 by controlling histone deacetylases, which results in increased Rac1 activation [55]. Rac1 mediates the overproduction of ROS, which in turn triggers NF-κB activation and, ultimately, TNF-α release [55]. AGEs also upregulate the expression of long non-coding RNA MALAT1, a competing endogenous RNA targeting MCP-1 by binding miR-124, which directly regulates MCP-1 expression by targeting the 3’-UTR promoter of MCP-1 [59].

#### Free fatty acids

Free fatty acids are a relevant link between obesity, insulin resistance, and type 2 diabetes (T2D). T2D patients have elevated free fatty acid levels in the blood and some studies have used palmitate, one of the most abundant saturated fatty acids in nature, to simulate diabetic conditions in vitro. Palmitic acid treatment triggers lipotoxicity in microglia: reducing cell viability [60–62], increasing fragmented, condensing nuclei [61] and promoting apoptosis via upregulation of cleaved caspase-3 and Bax proteins [63]. Besides, palmitate-induced metabolic stress impairs mitochondrial function (decreased mitochondrial mass and mitochondrial membrane potential), exacerbates oxidative stress and ROS production (peroxide, superoxide and NO) [16, 60, 61], decreases superoxide dismutase expression and elicits biomolecules oxidation [61]. It was initially reported that palmitate treatment decreases TNF-α expression and concomitantly upregulates arginase-1 and chitinase 3 [64], IL-4 and IL-10 [16]. However, others reported that palmitate exposure augments Iba1 expression [63], triggers a morphological shift toward amoeboid shape and increases microglial proliferation [16]. Moreover, palmitate treatment upregulates the expression of the pro-inflammatory cytokines IL-1β, IL-6 and TNF-α from 12 h onward in a dose-dependent manner [60], with the maximum production observed at 24 h for IL-1α, IL-1β, IL-2, IL-3, IL-9, IL-23, TNF-α and IFN-γ [16], with a concomitant overexpression of TLR4 [62]. In line with this, palmitate stimulation induces microglial responses through the TLR4/Akt/mTOR signaling pathway [63], which might involve free fatty acid-mediated metabolic reprogramming toward lipids utilization, as has been described for other immune cell types [65]. Accordingly, palmitate-induced metabolic stress activated the NF-κB pathway at different levels: decreased IκBα and increased p65 protein expression [61], augmented p65 phosphorylation [60], increased p65 translocation into the nucleus [60, 61] and enhanced NF-κB transcriptional activity [60]. Microglia treatment with ketone bodies, mimicking diabetic ketoacidosis, decreases viability in a dose-dependent manner by inducing apoptotic cell death [66]. Similar to palmitate, ketone bodies induce oxidative stress by promoting ROS generation and upregulating superoxide production in mitochondria, and also trigger the expression of cytokines (acute treatment upregulates IL-1β, IL-6, TNF-α, IL-4, IL-10 and IL-13, but longer exposure only upregulates IL-1β and IL-6) [66].

As diabetes is characterized by both hyperglycemia and hyperlipidemia, one study simulated diabetic conditions by incubating microglial cells in culture medium supplemented with HG and free fatty acids (mixture of palmitic and oleic acid) [67]. HG-free fatty acids combined treatment augments Iba1 and CD11b expression and triggers a morphological shift from ramified to amoeboid shape, elicits oxidative stress (upregulation of iNOS, cyclooxygenase-2, ROS and NO), and increases the secretion of IL-1β and TNF-α [67].

### Preclinical models

**Fig. 3:**
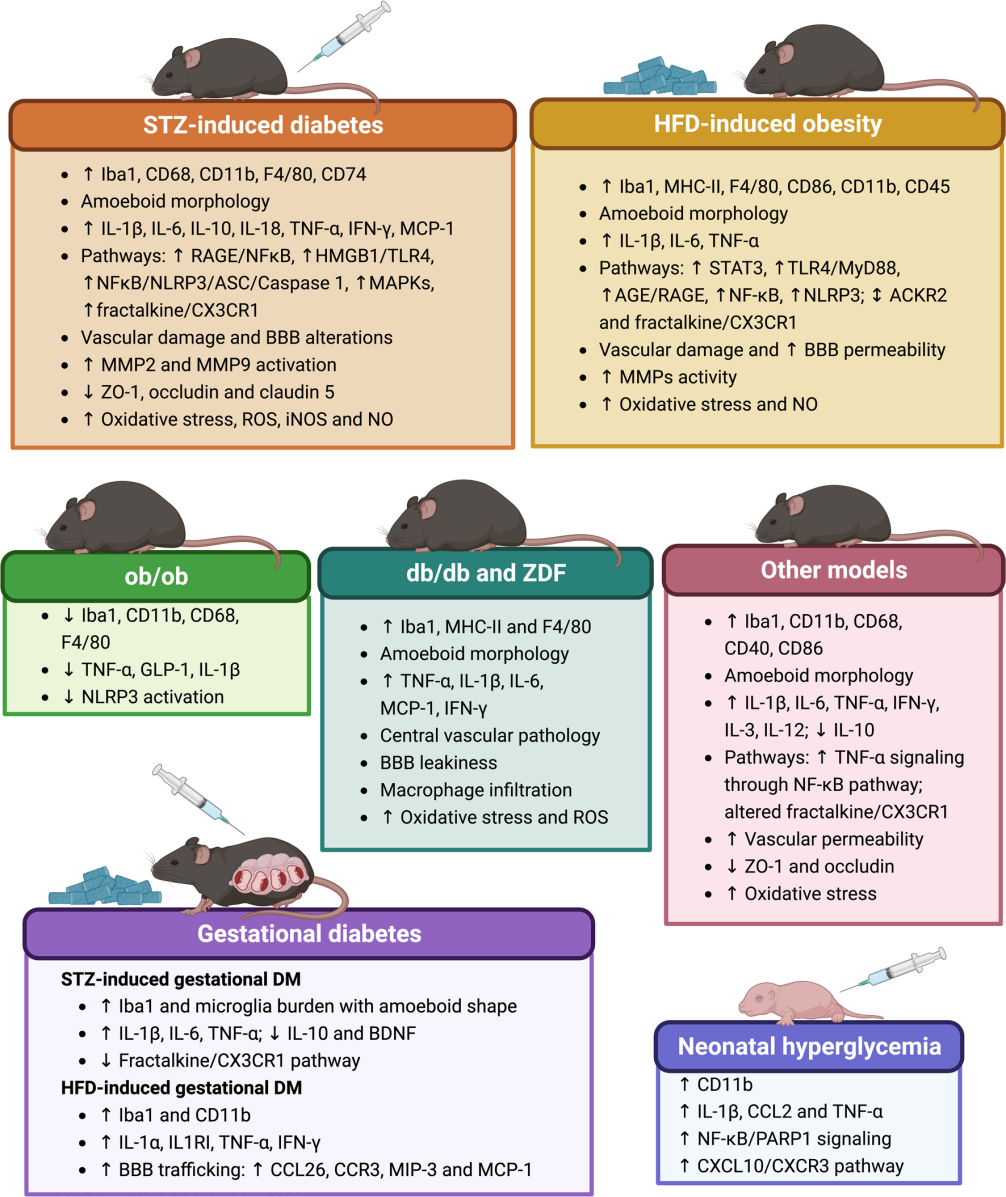
Modulation of microglial physiology in preclinical models of diabetes. Summarized responses of animal models of streptozotocin-induced diabetes, high fat diet-induced obesity, ob/ob mice, db/db mice, Zucker diabetic fatty rats, other models (insulin-induced hypoglycemia, β-pancreatic cell dysfunction, Ins2^Akita^ and Akimba mice, non-obese diabetic mice, spontaneous type 2 diabetes)**,** gestational diabetes and neonatal hyperglycemia are summarized

Research carried out in experimental animal models can also contribute to a better understanding of the inflammatory complications involved in metabolic-induced neurodegeneration. In this section, we will address the main results of diabetes-induced microglial phenotypes described in a variety of preclinical models of DM. A summary of this information can be found in Fig. 3.

#### Streptozotocin (STZ)-induced diabetic mice

STZ is an antineoplastic drug, produced by *Streptomyces achromogenes*, approved by the Food and Drug Administration in 1982 to treat metastatic cancers of pancreatic islets of Langerhans [68]. Given its capacity to selectively destroy β-pancreatic cells, STZ has been classically used to induce DM, specifically type 1 diabetes (T1D) in animal models by intravenous and, more commonly, by intraperitoneal administration, provoking insulin deficiency and hyperglycemia (for review [69]).

##### Inflammatory complications in the brain from animal models treated with STZ

Many previous studies have analyzed the neuroinflammatory process in the brain from animals treated with STZ, as a major complication associated with diabetes. A general increase in microglia population and related markers were observed in various brain regions. Some studies observed no differences in Iba1 levels from diabetic mice in the corpus callosum, thalamus, the piriform cortex [70], the basal nuclear region of the brain [71] or the substantia nigra, striatum and nucleus accumbens from rats treated with STZ [72, 73], or even reduced levels of Iba1 [74]. However, the majority of the studies reported a general increase of Iba1 in the brain from diabetic animals, including the cortex [71, 75–77], the hypothalamus [71] and the hippocampus from diabetic mice [63, 70, 75, 78–83] and rats [72, 73]. On the other hand, the levels of CD39, a transmembrane protein present on the surface of microglia that plays a critical role in preventing prothrombotic and pro-inflammatory effects, are reduced in the hypothalamus from diabetic mice [84].

Diabetes-associated inflammation has also been detected by other inflammatory markers. CD68 labeling shows that macrophages are increased in the hippocampus from STZ-treated rats [85] and mice, but not in the midbrain or the cerebellum, suggesting a region-specific vulnerability [79]. Likewise, CD11b labeling with OX42 increased in the hippocampus [85–87] and specifically in the hippocampal CA1 pyramidal area [88], as well as in the cortex and striatum [86, 89, 90], the pars nervosa of the pituitary [91], the hypothalamus, the basolateral amygdala [90] and the supraoptic nucleus [92] as diabetes progresses. The paraventricular nucleus [91] and the tractus solitarius also show increased presence of microglia by CD11b immunostaining, while the morphology of the cells is altered and they have fewer processes [93]. Likewise, CD11b is also increased in the hippocampus and the cortex from young rats, especially in diabetic ketoacidosis [94]. MHC-II labeling shows microglia with amoeboid morphology in the hippocampus [72] and pars nervosa of the pituitary from diabetic rats [91] and lipocalin-2, an acute phase protein that promotes neuroinflammation by eliciting microglia inflammatory responses, is increased in the hippocampus from mice treated with STZ [78]. STZ-treated mice also show reduced fractalkine mRNA and protein levels in the hippocampus [95].

Although it has been shown that IL-6 and IL-12 mRNA levels are not affected in the brains of diabetic mice [71], the vast majority of studies have reported that mRNA and protein levels of cytokines including IL-1β, IL-6, IL-10, IL-18, IFN-γ, MCP-1 and TNF-α are largely increased in the brain [30, 63, 71, 76, 78–80, 85, 87, 96, 97] and cerebrospinal fluid [87] from diabetic rodents. Interestingly, levels of IL-10 and IL-12p70 in brain lysates are significantly elevated in rats previously exposed to diabetes ketoacidosis when compared with diabetic rats alone [98].

Inflammation at the brain level also results in vascular damage and alterations of the blood–brain barrier (BBB). Inflammation and vascular injury in the hypothalamus from diabetic mice might be mediated by matrix metalloproteinase-2 activation and indoleamine 2,3-dioxygenase production [84]. BBB compromise in STZ-treated animals results in reduction of zona occludens-1 (ZO-1), occludin and claudin-5 in the hippocampus from diabetic rats [85].

Overall, an increase in oxidative stress markers are detected in brains from diabetic animals, directly associated with the inflammatory process. In this sense, malondialdehyde levels or mRNA levels of the NAD(P)H oxidase components, *gp91phox* and *p22phox* are increased after STZ-induced diabetes [76, 96]. ROS and malondialdehyde levels are increased in the prefrontal cortex [77] and the hippocampus [79, 97] from diabetic mice, whereas the midbrain or cerebellum seem to be spared [79]. On the other hand, antioxidant markers are reduced and both glutathione and glutathione peroxidase 4 are lower in the prefrontal cortex [77], while superoxide dismutase activity is limited in the hippocampus [97] from diabetic mice. Likewise, higher lipid peroxidation (measured by 4-hydroxy-2-nonenal immunostaining) supports an increase in oxidative stress in the hippocampus from diabetic rats [88] and a gradual increase in nitrate–nitrite levels are observed in the brains from diabetic mice [90]. Similarly, iNOS is increased in the hippocampus from diabetic rats [85] as well as in the hypothalamus and cortex from diabetic mice [90]. In line with these observations, other studies in mice treated with STZ have observed a reduction of soluble RAGE. Its reduction has been associated with impaired endothelial function and BBB compromise as well as altered expression of genes involved in regulation of endothelial functions and inflammation [99]. In addition, activation of RAGE/NF-κB signaling is observed in the hippocampus from mice treated with STZ [80]. Other studies have shown similar outcomes and increased phosphorylation of Iκβα, that stimulates the activation of NF-κB initiating the pro-inflammatory cascade, has been detected in the brain from diabetic mice [81]. A raise in phosphorylation of NF-κB p65 and IκBα is detected in the hippocampus from diabetic mice after STZ administration [63, 97] and higher NF-κB immunostaining is also detected in the hypothalamus, basolateral amygdala and cortex from diabetic mice [90]. Later studies have supported these observations showing that altered signaling in the hippocampus from diabetic mice includes increased protein expression of HMGB1/TLR4/NF-κB pathway, NLRP3 inflammasome [97] and the glucocorticoid receptor/NF-κB/NLRP3/apoptosis-associated speck-like protein (ASC)/caspase-1 pathway [100].

Other studies have induced diabetes in knockout (KO) mice to assess the specific role of the ablated protein in the inflammatory process. In this sense, formyl peptide receptors ablated (*Fpr2*^−/−^) mice treated with STZ have reduced activation of vascular endothelial cells and glia, with reduced production of pro-inflammatory cytokines [101]. In addition, diabetic lipocalin-2 KO mice show diminished STZ-induced Iba1 overexpression by microglia, limited macrophage infiltration and decreased pro-inflammatory cytokines expression including TNF-α and IL-6, supporting a role for lipocalin-2 in diabetes-induced brain inflammation [78]. Similarly, *p66Shc*^−/−^ mice have less oxidative stress and, therefore, are resistant to diabetes-induced brain damage. Thus, when these mice receive STZ Iba1 immunostaining reveals no increase in this marker in cortical or hippocampal microglia [96].

##### Inflammatory complications in the retina from animal models treated with STZ

Extensive studies have also focused on the inflammatory process in the retina from STZ-treated animals, since diabetic retinopathy is one of the main complications associated with diabetes. It has been reported that retinal microglia under physiological conditions reside in the inner and outer plexiform layers, but STZ-induced diabetes promotes microglial migration into the neuronal or photoreceptor layers [52]. Both mRNA and protein levels of Iba1 are increased in retinal microglia from diabetic mice [21, 24, 25, 102–107] and rats [25, 38, 108–117]. Not only microglia numbers but also retinal microglia morphology is affected, showing fewer branches as well as fewer and shorter processes [117, 118], while endpoints are reduced and the number of microglia in contact with acellular capillaries increases [117].

Other inflammatory markers, such as F4/80, a cell surface glycoprotein expressed at high levels in infiltrating macrophages and microglia, are also increased in the retina from diabetic mice and rats [119–122] and the number of CD11b^+^ cells is also increased in the retina from diabetic rats [123]. CD11b labeling with OX42 has been used to analyze microglia phenotypes, and increased expression of CD11b and Iba1 can be observed in the retina as disease progresses, affecting the inner nuclear layer, the ganglion cell layer [111] as well as the inner plexiform layer [124]. CD11b immunostaining reveals not only increased signal in the retina from diabetic mice and rats, but also altered morphology with larger bodies and reduced processes [125–127], while TNF-α is increased in CD11b^+^ microglia cells in the inner retinal layer of diabetic rats [126]. Similarly, ED1 immunostaining, used as a pan-macrophage marker, is also increased in diabetic rats as disease progresses [116, 124] and CD74 is also increased in the retina from diabetic rats [128]. CD74, the MHC-II invariant chain implicated in different diseases sharing features of inflammation, is coexpressed with microglia, supporting the role of microglia as antigen presenting cell [127]. Isolectin B4 has also been used for microglia counting, showing an overall increase of retinal labeled cells in diabetic rats as disease progresses [129]. The expression of translocator protein 18 kDa, another microglia marker, is also increased in the retina from diabetic rats [114]. In line with these observations, a significant increase in the percentage of C–X3–C motif chemokine receptor 1 (CX3CR1)^+^/CD11b^+^ (macrophage/microglia) is also observed in diabetic mice [130]. The role of formyl peptide receptors has been addressed in diabetic retinopathy. These belong to the G‐protein‐coupled chemoattractant receptor family and are expressed by immune cells, including neutrophils, macrophages and microglia to mediate cell recruitment and release in response to pathogen‐ and tissue‐derived chemotactic agonists. STZ administration results in an upregulation of formyl peptide receptors in mouse diabetic retinas, especially in retinal vascular endothelial cells, in association with an increased number of microglia [101].

Although some studies have shown no changes in IL-6 and TNF-α levels in the retina from STZ diabetic rats [131], the mRNA expression as well as cytokine levels of TNF-α, IL-1β, IL-6, IL-18, M-CSF, CCL2 or CCL5 are largely increased in the retina from these models [23, 25, 38, 57, 59, 103–109, 112–115, 119, 121, 123, 125, 126, 130, 132–137]. Single cell RNA sequencing has corroborated these outcomes in the retinas from STZ-treated mice and showed that diabetes activates IL-17 signaling pathway in retinal vascular endothelial cells, increases the expression of inflammatory genes in retinal microglia and stimulates immediate early genes in astrocytes [138]. Specifically, M-CSF expression seems to be preferentially affected in the nerve fiber layer from diabetic retinas and immunoreactivity for the CSF receptor is observed in OX42^+^ microglia in the ganglion cell layer [132]. On the other hand, neuroprotective cytokines such as IL-4 and IL-10 or suppressor of cytokine signaling 3 [25, 125] seem to be reduced. Other studies have also revealed an increase in the pro-angiogenic SDF-1a receptor C–X–C motif chemokine receptor 4 in the retina from diabetic rats [108].

The inflammatory process is accompanied by increased oxidative/nitrosative stress [25, 38] and fluorescent dye dihydroethidium and nitrotyrosine immunofluorescent staining are elevated in the retinas from diabetic mice [105] as well as iNOS mRNA and protein levels [103, 109]. Other oxidative stress markers are also altered including increased levels of NO and malonaldehyde [123], AGE [127] or ROS levels [139], whereas glutathione content is reduced [123]. Similarly, Amadori-glycated albumin, the prominent form of circulating glycated proteins in vivo, is significantly increased after diabetes induction [112] and indoleamine 2,3-dioxygenase expression is elevated in CD39^+^ resident retinal microglia from STZ-treated rats, due to pro-inflammatory cytokines, especially IFN-γ [140]. RAGE mRNA expression is increased in the retina from diabetic mice as well as methylglyoxal, whereas retinal glyoxalase I enzymatic activity is reduced [120].

In this sense, described inflammatory processes also lead to changes in the blood–retina barrier (BRB) and different mediators have been described including AGEs [120]. Other studies have reported that the retinal increase of IL-1β in STZ-treated rats is limited to vascular endothelial cells [131], suggesting a preferential inflammatory damage of retinal vessels. ICAM-1 is increased in retinas from STZ-induced diabetic mice, mediating endothelium damage and leakage, ultimately contributing to BRB dysfunction in diabetes [103, 106, 109]. Some studies have reported no changes in claudin and ZO-1, or even an increase in retinal claudin-5 [106], although the majority of the studies show that tight junction proteins, including occludin, claudin-1, claudin-5, claudin-19 and ZO-1 are reduced in the retinas of STZ-induced diabetic rats and mice [21, 38, 104, 106, 107, 130, 135, 137, 139]. The BRB is also challenged in diabetes, as microglia respond to high glucose with a pro-inflammatory phenotype characterized by Iba1 overexpression, NF-κB activation, secretion of pro-inflammatory molecules (e.g., TNF-α) and chemokines (e.g., MCP-1 that can stimulate the arrival of leukocytes into retinal tissues, as observed in diabetic mice) [104]. This effect may be mediated by long non-coding RNA MALAT1, as it has been observed in STZ-treated rats [59]. Microglial matrix metalloproteinase-9 is also implicated in the destruction of BRB integrity and in regulating the inflammatory response [25]. Other studies have reported an increased expression of TNF-α and matrix metalloproteinase-9, required for inflammatory macrophage migration [25, 38, 105]. Microglia themselves have been described as a potential component of the BRB, and Agouti rats treated with STZ show an increase in microglia contact with retinal capillaries. They also show reduced retinal blood flow in response to short duration hyperglycemia, and this effect is mediated by the fractalkine/CX3CR1 signaling axis [141]. On the other hand, leukocyte adhesion to retinal vasculature (leukostasis—an indicator of low-grade chronic inflammation) is also increased in diabetic mice after STZ administration [120, 139]. This effect seems to be mediated by CD40, a TNF receptor superfamily member that regulates cellular and humoral immunity [142].

Different signaling pathways have been implicated in the retinal inflammatory process induced by STZ in vivo, including ERK1/2 and its upstream kinases c-Raf and MEK1/2 [21, 23, 24]. Other studies have reported increased p-ERK, p-JNK and p–p38 in the retina from STZ-treated mice [119], observing that activation of ERK and p38 in diabetes occurs in microglia and astrocytes [112]. In line with these observations, the TLR4/MyD88/NF-κB p65 signaling pathway [38, 106] is also activated in diabetic retinopathy. These changes are accompanied by increased nuclear accumulation of NF-κB p65, a major regulator of inflammation and transcription factor for regulating the expression of different pro-inflammatory factors and IκB kinase phosphorylation, an enzyme complex that is involved in propagating the cellular response to inflammation [104, 107]. These results have been confirmed in studies showing increased phosphorylation of p65, IκB, and IKK in diabetic retinopathy [107, 135]. Other studies also suggest the implication of the CD40/CD154 pathway; CD154 increase is detected in diabetes and CD40 upregulation is observed in retinal endothelial cells, Müller cells and microglia from diabetic mice, mediating vascular retinal damage [142, 143].

Other relevant approaches have included the administration of STZ to transgenic animals with different reporters to follow microglia and immune-related retinal cells, such as CX3CR1^GFP^ mice, that show green fluorescent microglia, permitting the visualization of retinal microglia phenotypic modulation and migration. After STZ administration, microglia migrate from the inner plexiform layer of the retina to the inner and outer nuclear layers, an effect mediated by an increase in aldose reductase [52, 116]. To further analyze the role of CX3CR1, other studies have compared the effect of STZ-induced diabetes in CX3CR1^GFP/GFP^, CX3CR1^+/GFP^ and CX3CR1^+/+^ mice showing that CX3CR1^GFP/GFP^ mice have more acellular capillaries (capillary-sized vessel tubes having no nuclei anywhere along their length), indicative of the vascular damage and increased apoptosis. These mice also have increased bone marrow macrophage accumulation in the retina when compared with diabetic wild-type (WT) animals. This supports that the absence of CX3CR1 recruits bone marrow derived immune populations into the retina, as well as reduced IL-10 expression, which is upregulated during clearance of diabetes-induced apoptotic cells. This suggests a predisposition for CX3CR1 deficient microglia to be less efficient in the resolution of inflammation [136]. To assess the direct role of CCL2 in diabetes-induced inflammation and vascular damage, CX3CR1^GFP^–CCL2^−/−^ mice have been generated and these animals show a significant reduction of amoeboid-shaped microglia and monocyte/macrophage infiltration [121]. Similarly, the role of Syk, a member of tyrosine kinase family implicated in transmitting signals from a variety of cell surface receptors to the cells, has been analyzed in Cx3cr1^CreERT2−EYFP/+^; Syk^fl/fl^ mice treated with STZ. Deletion of microglial Syk limits inflammation by reducing microglia density, maintaining the number and length of microglia processes and lowering mRNA and protein levels of TNF-α, IL-1β, B and CCL2 [19]. STZ administration to mice lacking RAGE has also revealed that the diabetic pro-inflammatory state is limited and F4/80 microglia are not increased in the retina from *Rage*^−/−^ mice. Retinal vascular damage and leukostasis were also ameliorated, supporting the role of oxidative stress and the RAGE in diabetic retinopathy [120]. Administration of STZ to class A scavenger receptor deficient mice (*Sr-a*^−/−^) results in a more severe version of diabetic retinopathy with increased inflammatory markers, including F4/80^+^ and CD64^+^ retinal populations and larger increases of pro-inflammatory cytokines, such as TNF-α, IL-1β, IL-6 or MCP-1, favoring an increase in pJNK/total JNK and pIκB/total IκB ratios, whereas pERK/total ERK ratio is reduced [119]. STZ has also been administered to netrin-4 KO mice, a laminin-related secreted protein and guidance molecule highly expressed in the basal membrane of the retinal vasculature which might contribute to vascular stabilization and modulate the inflammatory process [144]. Netrin-4 KO mice show a significantly higher number of amoeboid-like cells in the retina and STZ-induced diabetes contributes to maintaining this phenotype, accompanied by higher mRNA expression of ICAM-1, CCL-2 and IL-6, suggesting that netrin-4 KO mice have a more robust pro-inflammatory profile and that netrin-4 plays a protective role against inflammation in the diabetic retina [144]. The inflammatory response is also increased in diabetic mice lacking A2A adenosine receptor, showing a significant increase of TNF-α in the retina [134]. In addition, STZ administration to transgenic mice that express CD40 in Müller cells results in upregulation of inflammatory markers and capillary damage. At the same time, TNF-α is upregulated in macrophages/microglia [143]. Other studies have analyzed the role of Nrf2, a key transcription factor involved in regulating oxidative stress, in Nrf2^−/−^ diabetic mice, showing its implication in the retinal inflammatory process and vascular damage [139].

#### High-fat diet-induced metabolic disease

High-fat diet (HFD) is commonly used to produce metabolic disorders in rodents to develop obesity, prediabetes and overt diabetes. Diet-induced obesity triggers low-grade chronic inflammation in peripheral tissues. This process is paralleled by a rapid response that activates signaling pathways producing inflammatory mediators that trigger glial cell accumulation in different CNS regions (reviewed by [145]).

##### Inflammatory complications in the brain from animals fed with HFD

It is well accepted that HFD is sufficient to promote neuroinflammation, mainly mediated by microglia (reviewed by [146]). Numerous studies have reported that HFD-fed animals show diet-induced expansion of microglial population in the hypothalamus [147–151] and nodose ganglion [148], as well as an accumulation of microglia compared to mice fed with standard-chow diet (SCD) in different brain regions including the cortex [152, 153], hippocampus [153–159], nodose ganglion [148, 160], hypothalamus [148, 149, 160–162] and arcuate nucleus of the hypothalamus [147, 150, 151, 163–170]. Conversely, a few studies found no differences in Iba1 expression between HFD rodents and SCD animals, either in the forebrain [171] or in the arcuate nucleus [172, 173]. Such discrepancies might be explained by variations in the composition and the duration of the diet (reviewed by [145]). Interestingly, diet-induced obesity alters the day/night rhythm of microglial activity in the medio basal hypothalamus, resulting in microglia with high activity and increased Iba1 expression during the whole day [164].

Besides Iba1, other markers of microglial responsiveness have been reported to be upregulated by HFD in rodents. The number of MHC-II^+^/Iba1^+^ cells is increased in the hippocampus of HFD-fed mice [154, 156], and the expression of the surface receptor F4/80 is augmented in the hypothalamus of HFD-fed rats [174] when compared to SCD-fed rodents, supporting a diet-induced pro-inflammatory microglial polarization profile. Furthermore, diet-responsive microglia express different markers in different hypothalamic regions, with CD169^+^ infiltrating macrophages assume a hybrid, microglia-like state [167]. Moreover, CD86 and F4/80 are overexpressed in the nodose ganglion of mice fed with HFD for only 1 day [148]. Interestingly, diet-induced microglial response is a reversible process that occurs after the development of obesity but prior to the onset of diabetes [154]. Such microglial plasticity is also supported by a time-course study performed in mice fed HFD for 1–28 days, in which hypothalamic microglia express different markers at different times, with an early increase in CD86 expression at 3 days on HFD, followed by a decrease in this pro-inflammatory marker concomitant with the overexpression of CD206 [175] and arginase-1 [176] at 7 days on HFD, and a final overexpression of MHC-II after 28 days on HFD [175]. Consistent with the upregulation of some microglial markers, many studies have reported that microglia from HFD-fed rodents display an amoeboid morphology with thickened processes in several brain regions [147, 150, 152, 153, 157, 165, 167, 168, 177]. Conversely, one study in which rats were exposed to free-choice high-fat high-sugar diet did not find morphological changes in microglia neither in the cortex nor in the hippocampus [178].

Pro-inflammatory cytokines and molecules, including IL-1β, IL-6, TNFα and Ikbkb, are largely upregulated in the brain of HFD-fed rodents compared to SCD animals [148, 150, 155, 156, 159, 160, 164, 166, 168, 174, 179, 180]. HFD-induced microglial phenotype is primarily a consequence of HFD itself, rather than obesity [165]. Contrary to inflammation in peripheral tissues, which appears over weeks or months after HFD exposure, hypothalamic inflammation in the arcuate nucleus develops as soon as within 1 day of HFD exposure, and follows an “on–off–on” pattern, characterized by a rapid onset of inflammation within the first days of HFD, followed by a neuroprotective response with a decline of inflammatory mediators, and posterior inflammation establishment by day 28 in HFD [147]. A similar hypothalamic microglial phenotypic switch within time was described in another study in which HFD feeding for 3 days triggered overexpression of pro-inflammatory markers (*Il1b*, *Il6* and *Cd74*), whereas 8 weeks on HFD resulted in a reduction in microglia-specific ‘sensing’ genes, including *P2ry12*, *Selplg*, *Slc2a5* and *Trem2*, accompanied by increased expression of *Pparg*, compared to microglia from SCD-fed mice [149], which evidences the temporality and plasticity of microglial responses to obesogenic diet.

The HFD-induced neuroinflammatory milieu described above is frequently accompanied by vascular damage and disturbance of the BBB. In the arcuate nucleus, prolonged HFD exposure results in an increased association of perivascular amoeboid-shaped microglia with microvessels, concomitant with augmented blood vessel length and diameter [151]. Activated leukocyte cell-adhesion molecule (ALCAM), expressed by endothelial cells and pericytes, has been postulated as a principal mediator of high-carbohydrate-HFD-induced angiopathy [181]. Signs of compromised BBB integrity have also been detected in rodents upon HFD exposure, including increased BBB permeability [151, 182] and extensive deposition of IgG inside hypothalamic microglia [163]. Possible triggers of BBB disruption in HFD-fed rodents are exacerbated proteolytic activity of matrix metalloproteinases due to an inflammatory-mediated imbalance between matrix metalloproteinases and metalloproteinase inhibitors [182], as well as NO overproduction [151]. A general increase in oxidative stress markers, closely associated with the inflammatory process, is detected in HFD-fed mice. Chronic exposure to HFD results in iNOS overexpression by hypothalamic microglia [151], increased lipid peroxidation and impaired mitochondrial activity (decreased oxidative phosphorylation complexes and diminished antioxidant enzymes, such as superoxide dismutase 1) [157].

Apart from the effects of HFD itself, other pathways are involved in HFD-induced neuroinflammation. Diet-induced obesity exacerbates leptin signaling, and this pathway plays a prominent role in HFD-induced hypothalamic responses, promoting an increase in the number of microglia [169]. Through their ability to orchestrate a complex inflammatory response upon HFD, microglia are critical regulators of the susceptibility to diet-induced obesity [166]. In fact, HFD induces activation of STAT3 specifically in microglia in the arcuate nucleus [183]. Interestingly, combined consumption of fat and sugar, but not only fat per se, triggers excessive AGEs production in hypothalamic neurons, as well as overexpression of RAGE by microglia. This leads to exacerbated microglial responses through the AGE/RAGE signaling pathway, including accumulation of microglia and morphological changes to amoeboid shape [181]. In line with this, dietary fats act through the activation of TLR4/MyD88 pathway in microglia, which induces endoplasmic reticulum stress as a downstream cascade that leads to hypothalamic inflammation [174]. IL-4 has been shown to exacerbate hypothalamic inflammation in HFD-fed rats through the IKKβ/NF-κB signaling pathway [176]. Other evidence supporting diet-induced activation of the NF-κB pathway in the hippocampus comes from the decreased expression of IκBα, a negative regulator of NF-κB, in HFD-fed mice [150]. Accordingly, the atypical chemokine receptor 2 (ACKR2), a negative regulator of inflammation due to its scavenger activity for inflammatory chemokines, is differentially regulated in the hypothalamus of obese-prone and obese-resistant Swiss mice early after HFD exposure, with obese-prone mice showing a smaller increment in ACKR2 than obese-resistant mice, indicating impaired restriction of inflammation [184]. Another relevant pathway in diet-induced early inflammation is the fractalkine/CX3CR1 pathway. A comparative study in which HFD was administered to various mouse strains with different predispositions to obesity revealed that fractalkine is the inflammatory marker with the most remarkable difference between strains [185]. Indeed, the obesity-prone Swiss mouse microglia produce TNF-α in response to fatty acids and promote the expression of fractalkine by neurons via TNF receptor 1 [185]. On the other hand, long-term HFD feeding mice show reduced expression of both fractalkine and CX3CR1 in the hippocampus and amygdala, accompanied by defective synaptic plasticity and cognitive impairment [186]. Until a few years ago, the majority of animal experiments were carried out without taking into account sex-differences. However, in recent years, it has been reported HFD differentially affects male and female rodents. Some examples of sex-differences in the response to HFD are increased microglia density (number of Iba1^+^ cells) [187, 188] and enhanced phagocytic activity (Iba1^+^/CD68 + cells) specifically in male microglia [188], whereas females showed no signs of HFD-mediated microglial phenotypic polarization [187, 188]. Consistent with such sex differences, fractalkine was increased in the hypothalamus of females on HFD, whereas male microglia showed decreased expression of CX3CR1 [187].

Besides affecting inflammatory pathways, obesogenic diet modifies microglial immunometabolism, triggering changes in metabolic substrate utilization, accompanied by altered rhythmicity of microglial circadian clock gene expression and disturbance of innate immune signaling [189]. Regarding metabolic substrate utilization by microglia, HFD decreases glucose and glutamate consumption while increasing lipid metabolism and energy production by mitochondria [189]. Indeed, prolonged exposure to HFD leads to excessive lipid accumulation in microglia, which suggests that these immune cells could uptake lipids for clearance and metabolic utilization [151]. In this way lipoprotein lipase, a key enzyme in cellular fuel uptake that delivers lipids by hydrolyzing triglyceride-rich lipoproteins, is overexpressed by hypothalamic microglia from mice exposed to high-carbohydrate-HFD from 3 days up to 10 weeks [190]. Likewise, uncoupling protein 2 (UCP2), a mitochondrial transporter that uncouples oxygen consumption from ATP synthesis, is transiently upregulated in hypothalamic microglia from HFD-fed mice, being overexpressed as soon as 3 days on the diet, restored at 7 days and further decreased by 8 weeks when compared to SCD-fed mice [191]. Finally, HFD has been reported to produce insulin resistance in the hippocampus [155, 156].

Recent studies about obesity-induced cognitive deficits have focused on the gut–brain axis, suggesting that microbiota dysbiosis is the primary response of the organism to hypercaloric diets, followed by neuroinflammation. In this regard, rats chronically fed with high-fat-sucrose diet develop gut microbiota dysbiosis and concomitant accumulation of amoeboid microglia in the prefrontal cortex [192]. Likewise, a time-course study on rats fed with high-fat-sucrose diet showed a diet-induced progressive remodeling of microbiota leading to microbiota dysbiosis, accompanied by increased Iba1 expression in the nucleus tractus solitarius as early as 4 weeks on the diet, and exacerbated at 26 weeks [193].

The effects of diet on central inflammation has also been reported in other models. Similar to the results obtained in rodents, high-glucose/high-cholesterol diet on zebrafish resulted in brain inflammation and CD11b overexpression, as well as elevated levels of IL-6 and TNF-α, and overexpression of the apoptotic genes caspase-3 and Bax [194]. Other studies have used genetically modified models fed with HFD to address the role of a specific protein on diet-induced neuroinflammation. Following this idea, TNF receptor 1 deficient mice on HFD overexpress TNF-α when compared to WT mice on the same diet, but no changes in downstream inflammatory markers were detected [185]. Conversely, IL-10 KO mice on HFD show increased expression of IL-1β, IL-6, TNFα, fractalkine and its receptor CX3CR1, suggesting a prominent role for IL-10 in dampening diet-induced neuroinflammation [185]. Mice lacking protein–tyrosine phosphatase 1B (PTP1B), a negative regulator of leptin signaling, have increased hypothalamic activation of the JAK2/STAT3 signaling pathway, as well as increased *Il10* and decreased *Tnfa* mRNA expression compared to WT mice on the same diet, indicating that PTP1B in microglia enhances inflammation by deactivation of the JAK2/STAT3 pathway [183]. Otherwise, lack of RAGE partially prevents the high-carbohydrate-HFD-induced increase of microglial number in the arcuate nucleus, indicating that microglia response to HFD is stimulated by other mechanisms apart from the AGE/RAGE pathway [181]. Expression of a functional TLR4 is required for HFD-induced hypothalamic infiltration of microglia, as well as microglia reactivity and expression of TNFα and IL-1β [185]. Conversely, TREM2 overexpression in the hippocampus of mice chronically fed with HFD prevents diet-induced microglial reactivity, including a shift in microglial polarization (overexpression of arginase-1 and YM1/2 markers, and decreased expression of iNOS2, TNFα, IL-1β and TLR-4) and the suppression of the HFD-mediated NF-κB pathway activation [195]. Moreover, specific deletion of IKKβ in microglial cells demonstrated that the IKKβ/NF-κB signaling pathway is necessary for HFD-induced hypothalamic microglial responses, including accumulation and morphological shift, as well as bone-marrow-derived CD169^+^ myeloid cells infiltration and accumulation [167]. Accordingly, mice deficient for TNF-α induced protein 3, a primary negative regulator of the NF-κB pathway, specifically in microglia lead to a heightened vulnerability to HFD-induced hypothalamic responses, including an increased number of microglia bearing morphological features of inflammatory phenotypes, as well as myeloid cell infiltration in response to HFD [167], which highlights the prominent role of NF-κB in HFD-mediated neuroinflammation. Overexpression of ACKR2, a cytokine decoy receptor, in the hypothalamus dampens diet-induced inflammation, including downregulation of microglial marker F4/80, as well as reductions in TNF-α, IL-1β and the chemotactic proteins MCP-1 and CX3CR1 [184]. In line with this, hypothalamic inhibition of fractalkine in mice on HFD results in decreased IL-1β burden and overexpression of CD206 by microglia [185]. Interestingly, female mice lacking CX3CR1 develop ‘male-like’ hypothalamic microglial expansion (accumulation of Iba1^+^ cells) and pro-inflammatory phenotype (overexpression of *Il6*, *Nfkbia* and *Ikbkb*), whereas central overexpression of CX3CL1 in male mice restrains HFD-induced microglial responses [187]. Other studies have addressed the role of proteins involved in stress responses. JNK1 ablation in HFD-fed mice showed a protective effect at the hippocampus, including decreased microglia number and size, and increased insulin sensitivity and antioxidant enzymes [157]. Mice lacking Nrf2, a pro-survival transcription factor that upregulates numerous genes involved in cellular resilience, show exacerbated HFD-induced oxidative stress, BBB disruption and concomitant increase in neuroinflammation in the hippocampus, mainly performed by Iba1^+^/CD68^+^ microglia that overexpress several pro-inflammatory cytokines and chemokines, compared to WT mice on the same diet [196]. Finally, relevant targets involved in microglial immunometabolism have also been described. Low-density lipoprotein receptor deficient mice show exacerbated HFD-induced microglia accumulation and amoeboid morphology in the hypothalamus compared to WT mice on the same diet [197]. Microglia-specific lipoprotein lipase knockdown mice on a high-carbohydrate-HFD show decreased Iba1 expression in the medio basal hypothalamus, as well as reduced microglial phagocytic capacity (CD68 expression) compared to control mice on the same diet [190]. Furthermore, lipoprotein lipase deficiency in microglia from mice on a high-carbohydrate-HFD results in mitochondrial dysfunction, including fewer and dysmorphic microglia when compared to WT mice on the same diet [190]. Other relevant mediator of HFD-induced changes in microglia is UCP2. Specific deletion of UCP2 in microglia prevented diet-induced stimulation of mitochondrial respiration, attenuates microglial accumulation and prevented the HFD-mediated morphological shift to amoeboid shape specifically in the arcuate nucleus [191]. Finally, deletion of calcineurin A beta, a calcium calmodulin-dependent phosphatase, in mice prevented HFD-induced increase in the number of microglia in the ventromedial hypothalamus [198].

##### Inflammatory complications in the retina from animal models fed with HFD

HFD-induced neuroinflammation has also been assessed in depth in the retina as a major complication of diabetic retinopathy (reviewed by [199]). HFD exposure to the rodent *Meriones shawi* triggered glial reactivity in the retina, including an increased microglial population and expansion from the inner to the outer retina [200]. In addition, HFD-fed mice exhibit increased CD11b^+^ and CD45^+^ macrophages in the ganglion cell layer, inner plexiform layer and outer plexiform layer of the retina [179], as well as microglia with amoeboid morphology [179, 201]. HFD-induced microglial phenotype in the retina results in IL-1β [201] and IL-6 [179] overexpression, as well as subsequent activation of the IL-6/STAT3 pathway, proposed as a key mediator of microglial recruitment across the retinal pigment epithelium cell layer, compromising BRB integrity [202]. In addition, HFD activates many inflammatory pathways, as is evident from TLR4 overexpression and increased activation of the NF-κB pathway [179], as well as the induction of inflammasome pathway, including overexpression of NLRP3, increased cleaved poly(ADP-ribose) polymerase-1 (PARP1), and augmented procaspase 1 and caspase 1 [201]. Concomitant with activation of inflammatory pathways, high-sucrose-HFD elicits oxidative stress, including peroxynitrite species and 4-hydroxynonenal, as well as overexpression of arginase 2 in the retina [201].

Transgenic models have allowed to confirm the prominent role of certain proteins in HFD-induced retinal inflammation. TLR4 deficient mice on HFD show decreased activation of the NF-κB-signaling pathway when compared to WT mice on the same diet [179], whereas arginase 2 deletion in mice prevents HFD-induced oxidative stress, microglial amoeboid shape and inflammasome signaling in the retina [201].

##### Inflammatory complications in animal models treated with HFD and STZ

Another experimental paradigm to mimic T2D in rodents consists of combining HFD feeding with STZ administration. A transcriptomic profiling of different tissue-resident macrophages, including microglia, revealed that the magnitude of the transcriptional response is higher in the STZ + HFD condition compared to HFD alone, suggesting a synergistic effect between diet and reduced β-pancreatic cell mass [203]. HFD-fed rodents administered with STZ have increased number of microglia in the hippocampus [204, 205], as well as overexpression of microglial activity markers CD11b and CD86 [206, 207]. Accordingly, rodents subjected to STZ + HFD treatment show general signs of neuroinflammation, including increased pro-inflammatory (IL-1β, IL-6, and TNF-α and NF-κB) and decreased anti-inflammatory (IL-10 and TGF-β1) factors in the hippocampus [207], as well as decreased expression of P2X4 receptors by microglia when compared to SCD rodents [206]. Furthermore, when compared to SCD rats, treatment with HFD and STZ induces RAGE overexpression in the hippocampus [204], where it mediates cytokine production and BBB abnormalities.

#### Leptin signaling-deficient rodents

##### *ob/ob* mice

The *ob/ob* mouse is a leptin-deficient monogenic mouse model of T2D, which has a spontaneous mutation in the leptin gene that impairs the secretion of bioactive leptin [208]. The *ob/ob* mouse is severely overweight, and while this might be a limitation of the model to study T2D strictly, it has been used for the last decades as a classical model to analyze the effects of diabetes [209].

It has been recently reported that *ob/ob* mice have apparent white matter lesions and microglial dysregulation (reduced levels of CD11b) in the hippocampus at 6 and 18 months of age [210, 211]. Accordingly, adult mice show decreased number of microglia and a tendency to reduced CD68 expression without changing the total number of microglia in the arcuate nucleus [165], aside from reduced TNF-α and GLP-1 expression in the frontal cortex [64] as compared with WT mice. The decreased expression of F4/80, NLRP3, Itga, Rab4a and IL-1β in *ob/ob* mice also supports that microglia activity is hindered upon leptin deficiency [165]. These observations might be due to the fact that leptin signaling also plays a role in microglia modulation, and therefore, the complete process is altered in *ob/ob* animals. Interestingly, when *ob/ob* mice are fed HFD for 2 weeks, the number of Iba1^+^ ramified processes and the expression of CD68 are increased compared to *ob/ob* SCD-fed mice, indicating that HFD per se can elicit microglial responses in the absence of leptin [165].

In the retina from *ob/ob* mice, microglia show increased expression of Iba1, deramification and a shift from dendritic to amoeboid morphology [212]. Microglia response in young obese mice is accompanied by increased leukostasis and early loss of retinal function, whereas microvascular damage, including loss of endothelial cells and increased vascular permeability, only occurs in older *ob/ob* mice [212].

##### *db/db* mice

The *db/db* mouse is a leptin receptor-deficient monogenic mouse model of T2D, in which a spontaneous mutation in the leptin receptor triggers a defect in leptin signaling. *db/db* mice have early onset obesity, hyperphagia and reduced energy expenditure [208].

No changes in microglia number are detected in young (6-week-old) *db/db* mice either in the cerebral cortex or the hippocampus [213], whereas older (14–36 weeks) *db/db* mice have increased number of microglia with augmented cell volume and amoeboid phenotype in comparison with WT mice in the same regions [213–219]. In the hypothalamus, *db/db* mice have similar numbers of microglia but with more ramified processes, as well as significantly lower transcription of *Atf3*, *Itgax*, *Rab4a*, *IL-1β* and *TNF-α* compared with WT mice [165]. Conversely, in the hippocampus *db/db* mice show more Iba1^+^ cells at different ages (8–36 weeks) [215, 216, 220–222], as well as overexpression of MHC-II, a marker of adaptative immune responses [221, 223], in comparison with WT littermates. Data supporting diabetes-induced microglial phenotype in *db/db* mice include IL-1β and TNF-α upregulation in the cortex [213] and hippocampus [220] of these mice; as well as higher secretion levels of IL-1β, IL-6, TNF-α and MCP-1 by isolated forebrain mononuclear cells [223]. Interestingly, ex vivo experiments with microglia isolated from *db/db* mice confirmed that diabetic microglia are primed and elicit a stronger response than WT microglia upon LPS stimulation [221]. Remarkably, gut microbiota is significantly different between WT and *db/db* mice, with diabetic mice having increased relative abundance of *Epsilonbacteraeota* and *Helicobacter*, both of which positively correlate with increased number of microglia and augmented TNF-α expression in the cortex and hippocampus [213]. *db/db* mice also present central vascular pathology, including exacerbated hemorrhagic burden [214–216] and BBB leakiness, which promotes parallel MCP-1 mediated macrophage infiltration and MHC-II expression by microglia [223]. In addition, invasion of amoeboid microglia into the neurovascular unit in *db/db* mice was also described [224]. These infiltrating microglia have an increased number of aberrant mitochondria, which might be responsible for their increased ROS production [224], and their somas have a shorter distance to the nearest microvessels compared to WT mice, which suggests hyperglycemia-induced, microglia-mediated vascular inflammation [219].

In retinas from *db/db* mice, arginase-1 expression is transiently elevated at 5 weeks of age, but returns to basal levels at 8 weeks, whereas iNOS is constantly elevated in parallel with disease progression [225]. At 10 weeks of age, *db/db* mice have a higher number of Iba1^+^ cells concomitant with increased apoptosis [226]. This pattern suggests a switch in microglial phenotype as disease progresses, including elevated levels of IL-1β, IL-6 and TNF-α in the retinas of 20-week-old *db/db* mice, which correlate with both increased serum levels of pro-inflammatory cytokines and progressive deterioration of visual function [225]. Studies in older mice confirmed such diabetic-induced microglial polarization, including induction of Iba1 and F4/80 in the retinas of diabetic mice by 24 weeks of age [227], and increased number of Iba1^+^ cells in 15-month-old *db/db* mice [228]. In addition, retinal microglia from *db/db* mice have morphological changes to amoeboid shape, as well as increased colocalization with isolectin B4, suggestive of a significant interaction between microglia and endothelial cells [228].

##### Zucker diabetic fatty (ZDF) rats

The Zucker diabetic fatty (ZDF) rat is an obese rat model of T2D that has a missense mutation in the leptin receptor, which leads to impaired leptin signal transduction, hyperphagia, impaired glucose tolerance and insulin sensitivity [208].

In comparison with Zucker lean rats, ZDF rats have an equal or even smaller microglial cell count in the hippocampus [229, 230] and in the arcuate nucleus of the hypothalamus [173]. However, some of these microglia are hypertrophied and have retracted processes suggestive of increased microglial reactivity, confirmed by augmented levels of IL-1β, IFN-γ, IL-6 and TNF-α [229, 230]. Signs of glia-induced neurodegeneration are also observed in the ZDF cortex and hippocampus, including decreased neuronal markers as well as amoeboid microglia [231]. Moreover, upregulation of oxidative stress hallmarks, including malondialdehyde or oxidized proteins, and concomitant decreased superoxide dismutase and glutathione peroxidase specific activities are seen in ZDF brains [231]. The BBB is also altered in ZDF rats, with overexpression of aquaporin 4 and age-dependent expression of GLUT1. This glucose transporter is downregulated in young diabetic rats, which could be a protective mechanism to avoid excessive glucose entry into neurons, whereas GLUT1 is overexpressed in older ZDF rats, which might be due to inflammation [231]. ZDF rats also show decreased retinal thickness when compared to Zucker lean animals, accompanied by increased number of microglia, although there are no evident signs of morphological changes [232].

#### Other models of DM

##### Insulin-induced hypoglycemia

Hypoglycemic brain injury, the most frequent acute complication of insulin-dependent DM (both T1D and advanced T2D), is mimicked in preclinical studies by intraperitoneal insulin injection in rodents. In vivo two-photon imaging of cortical microglia showed no effect of acute prolonged hypoglycemia (up to 90 min) in microglial function and motile surveillance, showing similar responses to normoglycemic microglia to laser-induced lesions, an effect attributed to metabolic switch to glutaminolysis to maintain surveillant behavior [39]. In the neocortex, rod-shaped microglia are transiently observed within an early period (3–6 h) after hypoglycemia, indicating microglial response to glucose deprivation. These cells are then replaced by hypertrophic amoeboid microglia (1–14 days post-hypoglycemia), which might be involved in phagocytosing irreversibly damaged neurons [233]. In the hippocampus, oxidative stress appears as early as 3 h after glucose deprivation with increased superoxide production [234, 235], resulting in decreased antioxidant glutathione and exacerbated oxidative injury at 7 days after the insult [236], time by which a severe pattern of neuronal death is evident [234–237]. Concomitantly, an increase in the number of CD11b^+^ cells is detected 24 h after hypoglycemia [237], reaching its peak at 7 days [235–237], and lasting up to 2 months after the induction of hypoglycemia [238]. In the long term, namely, 4 weeks after the hypoglycemic event, cell division is observed in the hippocampal CA1 region, with the majority of proliferating cells being CD11b^+^ microglia [238]. Recurrent/moderate hypoglycemic events also induce oxidative injury in hippocampal neurons, concomitant with increased CD11b expression and morphological shift in microglia toward amoeboid shape in the hippocampus [88] and the cortex [89]. Hypoglycemia-induced oxidative injury and microglia response are both mediated by superoxide production through NADPH oxidase activation, and are exacerbated in STZ-induced diabetic rats [88].

##### Other methods of β-pancreatic-cell dysfunction

Alloxan is a diabetogenic drug that has toxic effects on islets of Langerhans, but also affects other body organs, and it has been used as a model of T1D. A study analyzed microglial changes in alloxan-induced diabetic retinopathy in mice [239]. Three months after diabetes induction, alloxan-treated mice show neither loss of BRB integrity, nor neuronal apoptosis or macroglial reaction. Control and diabetic animals have similar number of microglial cells, but diabetic microglia show a morphological shift, including enlarged cell bodies and retracted processes [239].

On the other hand, transgenic rats overexpressing human amylin in the pancreas are a “humanized” model of hyperamylinemia that develop T2D-like alterations due to increased amylin-triggered β-cell apoptosis [240]. Chronic circulating hyperamylinemia promotes cerebral oligomeric amylin accumulation in 9-month-old diabetic rats. Such amyloid deposits elicit microglia-mediated neuroinflammation, as demonstrated by increased CD68^+^ amoeboid-like shaped microglia, clustered around blood vessels with amylin infiltration, accompanied by increased IL-6 and TNF-α and decreased IL-10 expression when compared to WT rats [240].

##### Ins2^Akita^ and Akimba mice

The Ins2^Akita^ mouse is a non-obese spontaneous T1D model that carries a spontaneous mutation in the *Ins2* gene, which triggers endoplasmic reticulum stress and ultimately leads to loss of pancreatic β-cells and hyperglycemia [208].

Ins2^Akita^ retinas show alterations in tight junction proteins, including downregulation of ZO-1 [241] and occludin expression [241, 242], as well as altered distribution of occludin in retinal blood vessels starting after 2 months of hyperglycemia [243]. Early pathological changes are followed by augmented retinal vascular permeability [242, 243] and subsequent appearance of acellular capillaries, as well as increased leukostasis [243]. Pathological angiogenesis is also observed in Ins2^Akita^ retinas, including upregulation of the pro-angiogenic VEGF [242, 244] and downregulation of the anti-angiogenic and neurotrophic pigment epithelium-derived factor [245]. Vascular lesions in diabetic mice are accompanied by morphological changes of both microglia, which show an amoeboid-like shape [241, 244, 245], and macroglia, with astrocytes losing contact with blood vessels in Ins2^Akita^ retinas [243]. In addition, microglia cell count [241] and Iba1 expression [241, 246], as well as Iba1^+^/CD68^+^ microglia [242] are increased in diabetic retinas, concomitant with an increase in numerous cytokines including IFN-γ, IL-6, IL-3, IL-12 and TNF-α [241], aside from alterations in retinal microglia arrangement [247]. Furthermore, Ins2^Akita^ retinas overexpress fractalkine [244], which could exert a protective effect on retinal vasculature [247], and consequently disruption of the fractalkine/CX3CR1 signaling in Ins2^Akita^ CX3CR1-deficient mice results in dysregulated retinal microglial responses, including increased microglial cell numbers and exacerbated inflammatory response [244], besides morphological changes to amoeboid-like shape [248]. Contrary to the increased vascular permeability described in diabetic retinas, neither pericyte coverage of blood vessels nor BBB integrity are affected by chronic hyperglycemia in Ins2^Akita^ brains [248].

The Akimba mouse is a double transgenic mouse model (Ins^2Akita^ x VEGF^±^) that combines *Ins2* mutation with a transient photoreceptor-specific overexpression of human VEGF, and thus presents hyperglycemia, vascular hyperpermeability and neovascularization in the retina. Akimba mice show characteristic features of advanced stages of diabetic retinopathy, including neurodegenerative retina and decreased relative blood flow volume, accompanied by an augment in Iba1^+^/OX42^+^ microglia, as well as retinal infiltration of perivascular macrophages (F4/80^+^/CD14^+^) through a leaky BRB [249]. This immune response is accompanied by increased IL-1β and IL-6 expression, concomitant with NLRP3 inflammasome activation (upregulation of NLRP3, ASC and activated caspase-1) [249]. A single-cell transcriptomic analysis of the retina from Akimba mice revealed that the diabetic retina is dysfunctional, having increased isolectin B4^+^ microglia/macrophages, with increased activation of immune-related pathways, and infiltration of immune cells [250].

##### Non-obese diabetic (NOD) mice

The non-obese diabetic (NOD) mouse is a model for T1D which develops spontaneous autoimmune diabetes due to exacerbated reaction of pancreatic macrophages to inflammatory stimuli. Prediabetic NOD mice had a higher microglial proliferation rate and overexpressed the co-stimulatory markers CD40 and CD86 when compared to non-diabetic CD1 mice [251]. Transcriptomic analysis on purified microglia from NOD and CD1 mice revealed that the main differences are detected in cellular functions related to cell cycle and cell survival, without any evidence of prediabetes-induced inflammation. However, systemic LPS administration to NOD mice elicited an exacerbated and prolonged brain immune response compared to CD1 mice, including increased microglial proliferation, augmented pro-inflammatory mediators production and activation of cellular functions related with cell-to-cell signaling, supporting microglial priming induced by a prediabetic state [251].

##### Models of spontaneous T2D

The Goto Kakizaki rat is a Wistar non-obese model for spontaneous T2D that develops glucose intolerance due to insulin resistance. In the cerebral cortex, hyperglycemia specifically affects microglial population, and 13-month-old Goto Kakizaki rats have less microglia, but of larger average volume compared to Wistar rats [252]. Likewise, diabetic rats have higher retinal Iba1 staining, as well as some hypertrophic cells, compared to non-diabetic rats at 7 [253] and 12 [254, 255] months of age. Furthermore, the retinas of Goto Kakizaki rats show macrophage infiltration (increase in CD11b^+^ cells) accompanied by vascular changes (increased tortuosity of microvessels and more blood vessels) [253]. Chronic hyperglycemia also disrupts cell junctions between retinal pigment epithelial cells, indicative of BRB breakdown, and alters the physiological transcellular trafficking of microglia through retinal pigment epithelial cells, triggering the accumulation of microglia in the subretinal space [254].

The KK-Ay model is a Kuo Kondo mouse bearing the yellow obese gene that spontaneously develops severe obesity, hyperglycemia and insulin resistance. Similar to other diabetic models, KK-Ay mice show gradual microglial phenotypic modulation in the hippocampus with aging (5–7 months), including increased Iba1 immunoreactivity concomitant with amoeboid-like morphology, as well as overexpression of IL-1β and TNF-α [256].

One study performed single-cell transcriptomics on the retina of macaques with spontaneous T2D and characterized the interactome of the diabetic retina [257]. Microglia showed the highest susceptibility to hyperglycemia among the six major retinal cell types. Hyperglycemia triggers microglial responses through enhanced autocrine TNF-α signaling, which activates NF-κB and its downstream inflammatory genes. Conversely, cell-to-cell communication between microglia and other retinal cells is a potential feedback mechanism to restrain exacerbated microglial inflammatory responses under hyperglycemia, including microglial interactions with Müller cells (through osteopontin), amacrine cells (mediated by growth arrest-specific protein 6) and cone photoreceptors (through transforming growth factor-β2) [257].

#### Neonatal diabetic models

##### Gestational DM

Gestational DM is a state of hyperglycemia and glucose intolerance that emerges during pregnancy and it is also associated with mild chronic systemic inflammation and elevated circulating free fatty acids. Various studies using different approaches to induce DM prior or during pregnancy have elucidated that the effects of hyperglycemia and/or hyperinsulinemia in an unfavorable intrauterine environment on offspring development.

T1D induction by STZ injection in rats at either pre-gestational [258] or gestational day 5 [259] does not modify hippocampal microglia phenotype in the offspring at an early neonatal period (postnatal days 1–21) [258, 259]. Conversely, the offspring from STZ-induced diabetic mouse mothers show early microglial response to maternal DM, including increased microglia number and size at postnatal day 7 [260]. This phenotype is sustained during early adulthood in pups from STZ-induced diabetic dams, which show increased microglia population with augmented size [260], as well as increased IL-1β, IL-6 and TNF-α production and decreased IL-10 and BDNF expression [259]. Furthermore, male offspring from STZ-induced diabetic dams have decreased microglial CX3CR1 expression, suggesting an impaired neuron–microglia communication through the fractalkine/CX3CR1 signaling [258].

Diet-induced maternal obesity cannot be exclusively identified as a model of DM. Despite this limitation, this approach is commonly used to mimic the effects of T2D and results in reduction of hippocampal CX3CR1 expression in the offspring [16], which might trigger deficits in synaptic pruning by microglia during postnatal development. In line with these observations, this model also elicits early hippocampal neuroinflammation in the offspring (at embryonic day 20), including an increased proportion of microglia showing an amoeboid morphology and overexpression of IFN-γ, IL-1α, TNF-α and MCP-1 [16]. Conversely, another study using Tet29 animals, a transgenic rat model of T2D in which the insulin receptor is knocked down via doxycycline-induced RNA interference, reported that, at gestational day 21, pups have reduced Iba1 expression in the hippocampus compared to pups from normoglycemic dams, but both groups showed similar microglial cell density [261]. Nevertheless, other studies have reported that gestational DM-induced neuroinflammation occurs early at postnatal days 1–20 (increased CD11b, Iba1 and TLR4 expression) [262] and is maintained into young adulthood (15–20-week-old rats), including increased oxidative stress [263] and Iba1 overexpression by microglia [16, 263]. Interestingly, the offspring from obese dams show microglia priming and exacerbated inflammatory response to intraperitoneal LPS challenge, with overexpression of Iba1 and IL-1β [262]. In line with this, a study on female primates fed with HFD for 4 years revealed that maternal obesity triggers a neuroinflammatory response in fetal hypothalamus, including upregulation of IL-1 receptor type 1, mainly expressed by endothelial cells of the microvasculature, and increased Iba1 expression, accompanied by elevated levels of IL-1β and molecules involved in BBB trafficking, such as CCL26, CCR3, macrophage inflammatory protein (MIP)-3 and MCP-1 [264].

##### Neonatal hyperglycemia

Neonatal hyperglycemia is a common complication in extremely low-birth-weight preterm infants, associated with increased risk of mortality and brain damage. Although it cannot be classified as T1D or T2D, neonatal hyperglycemia (induced by injecting STZ on postnatal day 2 rats) increases the number of CD11b^+^ microglia in the hippocampal CA1 region at postnatal day 6 and triggers long-term deficits in learning and memory and decreased dendritic density in 3-month-old rats [265]. In addition, hyperglycemia enhances NF-κB signaling pathway—increased NF-κB and its co-activator PARP1 expression—and also promotes the overexpression of CXCL10 by neurons, microglia and astrocytes, and its receptor CXCR3, expressed by neurons and microglia [265]. Similar results were reported in the cortex from a model of recurrent hypoinsulinemic hyperglycemia generated by repeated subcutaneous injection of octreotide and dextrose on postnatal days 3–12 in rats [266]. Hyperglycemia promotes a pro-inflammatory phenotype in microglia, including augmented number of CD11^+^ cells in the developing cortex, increased NF-κB and PARP1 expression and decreased IκB production [266].

Another study transiently induced hyperglycemia in newborn rat pups by STZ injection at postnatal day 1, resulting in a maintained augment on blood glucose levels from postnatal days 2–6 [267]. Microglial recruitment is transiently promoted in the retina of hyperglycemic pups at postnatal day 6, but it returns to basal levels after 14 days of normoglycemia. In addition, morphological signs of microglial response to hyperglycemia, as well as increased pro-inflammatory mediators, such as IL-1β, CCL2 and TNF-α, were described [267].

### Clinical studies

Most of the knowledge about the effects of diabetes on microglial physiology has been obtained from cell culture research (from seminal work in the early 2000s [10, 56] to recent articles [9, 42, 63]), as well as studies on well-established preclinical models of diabetes (for review on these models, see [208]). However, in the recent years, various clinical observational studies have confirmed basic research results, providing important insights into how diabetes affects microglia in humans. In this section, we will summarize both in vivo and *post-mortem* studies in human samples.

#### In vivo

Various studies analyzing different biomarkers in control and diabetic patients’ samples agree that diabetes promotes specific phenotypes in retinal microglia in vivo. Higher levels of soluble CD14, a cytokine expressed by microglia and associated with the immune response, are detected in the aqueous humor from diabetic patients when compared to control subjects [268]. In line with this, a case–control study reported an increase in inflammatory cytokines (IL-1β, IL-3, IFN-γ, IFN-γ-induced protein 10 and MCP-2) in the aqueous humor from diabetic subjects compared with non-diabetic samples [269]. Single-cell RNA sequencing on surgically harvested epiretinal fibrovascular membranes (one of the hallmarks of proliferative diabetic retinopathy) identified microglia as the main cell population in epiretinal membranes [270]. In a simplified approach, four microglia subclusters were described, corresponding to “reactive” microglia with profibrotic and fibrogenic properties, “anti-inflammatory” phenotype, “pre-activated” state and “proliferative” microglia. Compared to healthy microglia, “reactive” subtype overexpresses a subset of receptors involved in regulation of immune cell activation and mobilization (IFN-γ receptor, CD44, CD74, CCR1 and CCR5), whose ligands were upregulated in vitreous humor of diabetic patients [270]. Accordingly, a study comparing epiretinal membranes from idiopathic macular hole samples and diabetic patients reported overexpression of microglial activity markers (Iba1, CD11b and F4/80) and exacerbation of inflammatory mediators (IL-1β, VEGF and matrix metalloproteinase-9) in diabetic retinas, and such suggested that the microglial response is oxidative-stress mediated, as reflected by the upregulation of oxidative stress markers (HIF-1α and Nrf2) in diabetic samples [271]. Another study described increased concentrations of inflammatory and vasoactive biomarkers (including CCL5, MIP-1α, Fas ligand and VEGF), as well as decreased levels of osteopontin (modulator of microglial activity) and metallopeptidase inhibitors in the aqueous humor of diabetic patients, indicating not only deregulation of microglial phenotype but also extracellular matrix remodeling in diabetic retinas [272]. Outside the retina, a cross-sectional study reported elevated serum levels of the ectodomain of TREM2 in non-obese diabetic patients in comparison with control individuals, which correlates with increased risk of cognitive impairment [273]. Remarkably, the ectodomain of TREM2, derived from protease-mediated shedding, could be an indirect marker of microglial phenotype that crosses the diabetes-induced leaky BBB [273].

#### Post-mortem

Different *post-mortem* studies have confirmed microglial particular phenotypes in the retinas of diabetic patients. Microglial cell number is significantly increased in patients with diabetic retinopathy when compared with healthy retinas [274]. Moreover, in contrast with the ramified microglia found in healthy donors, diabetic microglia are hypertrophic and overexpress markers of activity at different stages of the disease, including HLA-DR, CD45 and CD68 [274], as well as CD74, Iba1, iNOS and arginase-1 [225]. Another study comparing both T1D and T2D diabetic retinas with non-diabetic controls reported increased number of Iba1^+^ cells, accompanied by decreased CD39 expression, which is only expressed by resident microglia, in diabetic retinas when compared with non-diabetic samples [140]. Microglial response also correlates with increased indoleamine 2,3-dioxygenase and quinolinic acid expression, as well as neuronal loss, in the retinas of diabetic patients [140]. The role of microglial connective tissue growth factor (CTGF) in the retina remains to be elucidated. One study reported a constitutive expression of CTGF in CD68^+^ microglia in control retinas, while CTGF was decreased in diabetic retinas [275]. Conversely, another study reported that CTGF is only expressed in microglia from diabetic retinas, while its expression is absent in microglia from healthy retinas [276]. These contradictory results suggest that microglia-derived CTGF might be involved in retinal homeostasis, although more studies are required to elucidate its precise role in the retina. Another molecule that has been described to be upregulated specifically in microglia only in diabetic retinas is adenosine deaminase-2 [277], which was reported as a target of miR-146b-3p [278]. In this regard, miR-146b-3p is downregulated, whereas adenosine deaminase-2 activity is enhanced in the vitreous humor of diabetic patients compared to healthy donors [278].

There are also a few post-mortem studies analyzing the brain of diabetic patients. Contrary to animal studies reporting increased hypothalamic microglial activity, no differences in Iba1-related parameters (soma number and size and ramifications) were detected between T2D patients and non-diabetic controls in the hypothalamic infundibular nucleus [279]. Likewise, similar number of microglial cells in the hypothalamic suprachiasmatic nucleus were reported in a study comparing tissue from T2D and non-diabetic individuals [280]. However, a positive correlation was described between body mass index and Iba1^+^ soma size, suggesting an association between obesity and microglial reactivity [279]. In line with this, microglia response to obesity is evident in the hypothalamus of patients with body mass index > 30, having increased number of Iba1^+^ cells and with amoeboid morphology when compared to brain tissue from normal weight individuals [149]. Otherwise, the cerebral cortex from obese patients had no signs of microglial reactivity [149], but presented with an increased number of CD45^+^ immune cells, paralleled by CD31 and laminin reductions, indicative of possible leukocyte transmigration through a leaky BBB [182].

## Concluding remarks

Evidence analyzed in the present systematic review suggest that diabetes and its related conditions have a profound impact on microglial physiology, triggering a wide variety of microglial responses. Despite different methodologies and experimental paradigms, in vitro studies consistently reflect that diabetes-related conditions—namely, high d-glucose, aglycemia, changes in glucose levels, AGEs exposure and elevated concentration of free fatty acids—have a profound impact on microglial phenotype, triggering the upregulation of certain microglial activity markers, such as Iba1 and several receptors like CD11b, CD68 and MHC-II, concomitant with a morphological shift into amoeboid shape and the release of a plethora of cytokines and chemokines involved in intercellular communication, including IL-1β, IL-6, TNF-α (pro-inflammatory mediators), M-CSF (regulator of microglia proliferation), MCP-1 (regulator of monocytes migration) and VEGF (a pro-angiogenic factor). A generalized increase in oxidative stress (augmented ROS and NO production) is observed when microglia are exposed to diabetes-related conditions, accompanied by the activation of several pathways, comprising inflammatory pathways (NF-κB and NLRP3 inflammasome), oxidative stress-related pathways (HIF-1α), mitogen-activated protein kinases responding to growth factors (MEK/ERK), inflammatory cytokines (p38) and stress (JNK), the AGE/RAGE pathway, as well as the Akt/mTOR pathway, involved in regulating the cell cycle and also metabolic reprogramming. In fact, microglia show a unique metabolic flexibility that allows them to utilize alternative substrates, such as glutamine, during glucose deprivation, or lipids, when exposed to an excess of free fatty acids, allowing them to maintain their surveillant functions. Preclinical models of diabetes add another layer of complexity, since microglial cells, with their inherent plasticity and local heterogeneity, interact with other cell types in a more physiological but also complex environment. A wide variety of genetic, experimentally induced and diet-induced models of diabetes confirm the in vitro evidence about diabetes-mediated modulation of microglial states, showing a generalized amoeboid morphology accompanied by the overexpression of activity markers. Besides Iba1 the majority (CD11b, CD68, F4/80, CD74, MHC-II, CD86, CD45, CD40) are surface receptors, supporting the increased interactions of microglia with the diabetic environment. Studies in animal models have also revealed that the fractalkine/CX3CR1 pathway is stimulated, reflecting close interactions between neurons and microglia. Diabetic models also show central vascular pathology and BBB permeability, probably mediated by oxidative stress, accompanied by macrophage infiltration into the CNS. Remarkably, gestational DM models have reported that the offspring from diabetic dams have a specific microglia signature characterized by overexpression of Iba1 and CD11b, overproduction of IL-1β, IL-6, TNF-α, increased NF-κB activation and decreased fractalkine/CX3CR1 signaling. Studies modeling neonatal hyperglycemia in rats have reported similar effects, reflecting that diabetes can modulate microglia states both during development and early after birth, and such diabetes-induced microglia phenotype is maintained long-term. Finally, clinical studies have also reported diabetes-mediated modulation in human microglia. In this regard, microglia from diabetic patients are hypertrophic and overexpress several activity markers, including the receptors CD11b, CD44, CD45, CD68, CCR1 and CCR5, as well as Iba1, iNOS and arginase 1, as well as pro-inflammatory and vasoactive biomarkers, such as IL-1β, IFN-γ, IL-3, CCL5, MIP-1α, Fas ligands and VEGF. Overexpression of oxidative stress markers such as HIF-1α and Nrf2 is accompanied by decreased expression of CD31 and laminin, indicative of a leaky BBB.

The comprehensive picture emerging from this systematic review summarizes the current knowledge about the role of diabetes and its related conditions as critical modulators of microglial phenotypic states, and it should help to the development of new treatments for metabolic-induced cognitive decline with an inflammatory component by targeting microglial cells.

## Supplementary Information

**Additional file 1.** Complete Review Library.

## Data Availability

Not applicable.

## Abbreviations

ACKR2: Atypical chemokine receptor 2
AGEs: Advanced glycation-end products
ALCAM: Activated leukocyte cell adhesion molecule
ASC: Apoptosis-associated speck-like protein
ASK1: Apoptosis signal-regulating kinase 1
BBB: Blood–brain barrier
BRB: Blood–retina barrier
CCL2: Chemokine (C–C motif) ligand 2 (also referred to as MCP-1)
CCR: C–C motif chemokine receptor
CD: Cluster of differentiation
CNS: Central nervous system
CSF1R: Colony stimulating factor 1 receptor
CTGF: Connective tissue growth factor
CX3CR1: C–X3–C motif chemokine receptor 1
DM: Diabetes mellitus
ERK: Extracellular signal-regulated kinase
GLUT1: Glucose transporter type 1
GLUT2: Glucose transporter type 2
GLUT5: Glucose transporter type 5
HFD: High-fat diet
HG: High d-glucose
HIF-1α: Hypoxia-inducible factor 1-alpha
HMGB1: High mobility group box 1 protein
Iba1: Ionized calcium-binding adapter molecule 1
ICAM-1: Intercellular adhesion molecule 1
IFN-γ: Interferon-gamma
iNOS: Inducible nitric oxide synthase
IRAK1: Interleukin 1 receptor-associated kinase 1
JAK2: Janus kinase 2
JNK: Jun N-terminal kinase
KO: Knockout
LPS: Lipopolysaccharide
M-CSF: Macrophage colony-stimulator factor
MALAT1: Metastasis-associated lung adenocarcinoma transcript 1
MAPK: Mitogen-activated protein kinase
MCP-1: Monocyte chemoattractant protein-1
MEK: Mitogen-activated protein kinase kinase
MHC-II: Major histocompatibility complex II
MIP: Macrophage inflammatory protein
mTOR: Mammalian target of rapamycin
MyD88: Myeloid differentiation primary response 88
NF-κB: Nuclear factor kappa B
NG: Normal glucose
NLRP3: NLR family pyrin domain containing 3
NO: Nitric oxide
NOD: Non-obese diabetic
Nrf2: Nuclear factor erythroid 2-related factor 2
PARP1: Poly(ADP-ribose) polymerase-1
PTP1B: Protein–tyrosine phosphatase 1B
RAGE: Receptor for advanced glycation end products
ROS: Reactive oxygen species
SCD: Standard chow diet
STAT3: Signal transducer and activator of transcription 3
STZ: Streptozotocin
Syk: Spleen tyrosine kinase
T1D: Type 1 diabetes
T2D: Type 2 diabetes
TLR4: Toll-like receptor 4
TNF-α: Tumor necrosis factor alpha
TRAF6: Tumor necrosis factor receptor-associated factor 6
TREM2: Triggering receptor expressed on myeloid cells 2
UCP2: Uncoupling protein 2
VEGF: Vascular endothelial growth factor
WT: Wild type
ZDF: Zucker diabetic fatty
ZO-1: Zona occludens-1
